# Potential Role of Intracranial Mast Cells in Neuroinflammation and Neuropathology Associated with Food Allergy

**DOI:** 10.3390/cells11040738

**Published:** 2022-02-20

**Authors:** Danielle L. Germundson, Kumi Nagamoto-Combs

**Affiliations:** 1 Clinical and Translational Sciences Graduate Program, University of North Dakota School of Medicine & Health Sciences, Grand Forks, ND 58202-9037, USA; danielle.germundso.1@und.edu; 2 Department of Biomedical Sciences, University of North Dakota School of Medicine & Health Sciences, Grand Forks, ND 58202-9037, USA

**Keywords:** beta-lactoglobulin, blood–brain barrier, cow’s milk allergy, demyelination, histamine, IgE, IgG, neuroinflammation, proteases, astrocyte

## Abstract

Mast cells (MCs) are the major effector cells of allergic responses and reside throughout the body, including in the brain and meninges. Previously, we showed in a mouse model of subclinical cow’s milk allergy that brain MC numbers were elevated in sensitized mice. However, the neurophysiological consequences of intracranial MC accumulation and activation are unclear. We hypothesized that centrally recruited MCs in sensitized mice could be activated by the allergen via the IgE/FcεRI mechanism and increase the blood–brain barrier (BBB) permeability to promote neuroinflammation. Furthermore, we suspected that repeated allergen exposure could sustain MC activation. To investigate our hypothesis, we sensitized C57BL6/J mice to a bovine whey allergen, β-lactoglobulin (BLG), and subsequently placed them on a whey-containing diet for two weeks. MC activity and associated changes in the brain were examined. BLG-sensitized mice showed mobility changes and depression-like behavior with significantly increased MC numbers and histamine levels in select brain regions. IgG extravasation and perivascular astrogliosis were also evident. Importantly, myelin staining revealed cortical demyelination in the BLG-sensitized mice, suggesting a potential neural substrate for their behavioral changes. Our findings support the ability of brain MCs to release histamine and other mediators to increase BBB permeability and facilitate neuroinflammatory responses in the brain.

## 1. Introduction

Mast cells (MCs) are innate immune cells with secretory granules rich in various bioactive substances, including histamine [1], proteases [1], growth factors [2,3,4], cytokines [5,6,7], and chemokines [8]. Originating in the bone marrow, MC precursors travel through the blood and migrate into virtually every tissue of the body, where they then mature in a tissue-specific manner [9]. MCs are best studied in the context of type I hypersensitivity reactions, during which an allergen binds to specific IgE coupled with Fcε receptor I (FcεRI) on MC surfaces. This interaction causes rapid crosslinking of the IgE–FcεRI complexes followed by exocytosis of the MC vesicles, known as degranulation [10]. Releasing the preformed histamine and other proinflammatory substances from these vesicles causes immediate allergy symptoms, including erythema, pruritis, and edema. In addition, MCs are activated by various non-IgE-dependent stimuli, such as antigen-IgG complexes [11,12], toll-like receptor (TLR) ligands [13,14], cytokines [15], complement [16], and substance P [17] (for comprehensive reviews, see [18,19]). Activation of MCs by these substances causes complete degranulation or more selective release and de novo production of cytokines, chemokines, and secreted factors [18,19], which in turn promote pathogen clearance [13,14], regulate adaptive immune responses [20], suppress tumors [21], facilitate wound healing [22], and stimulate angiogenesis [23,24]. Thus, MCs are important for homeostasis as well as in pathologic conditions such as infections, cancers, and injuries.

MCs are also present in the central nervous system (CNS), including the brain parenchyma, choroid plexus, and meninges [25,26,27]. In the brain, MCs are located perivascularly on the brain side of the vasculature, particularly in the hippocampus, thalamus, and hypothalamus [26,28]. MCs have substantial physiological roles in the CNS, contributing a large percentage of histamine to the central pool [29] and regulating the blood–brain barrier (BBB) permeability [30]. Furthermore, MCs secrete factors that serve as neurotransmitters, growth factors, chemoattractants, and inflammatory mediators, interacting with neurons and glia through direct, paracrine, and transgranular communication [18]. These MC-derived factors influence synaptic transmission, neuroinflammation, and immune cell infiltration into the CNS, maintaining homeostasis. The absence of MC activities in MC-deficient mice or wild-type mice that have received intracerebroventricular administration of an MC stabilizer, cromolyn [31], is associated with the development of anxiety-like behavior, further supporting the role of MCs in the normal brain function.

Consequently, dysregulation of brain MCs is associated with various neuropathologies. The number of MCs rises after traumatic brain injuries or ischemic events, increasing BBB permeability to facilitate peripheral leukocyte influx and sustain the inflammatory state [32,33]. In post-mortem studies, MCs and their mediators have been observed around amyloid plaques [24] and in the demyelinating lesions of multiple sclerosis [34]. Furthermore, patients with conditions that present MC overactivity, such as an allergy or mastocytosis, often report anxiety, depression, and cognitive changes [35,36,37,38,39]. Rodent studies also support the involvement of MCs in neurological disorders. Increased numbers of MCs had been found in the hippocampus and cerebral cortex of APPswe/PS1dE9 mice, an Alzheimer’s disease mouse model, at least a month before amyloid plaques became detectable [40]. In contrast, the absence of MCs delayed the development of experimental autoimmune encephalomyelitis (EAE), a model of multiple sclerosis, and decreased disease severity in MC-deficient Kit^W/Wv^ mice [41]. These findings suggest that MCs accumulate and become activated in the CNS as one of the initial responders during the development of neurological disease and thus their activities may contribute to the pathogenesis of neuroinflammatory disorders and influence behavior.

Previously, our laboratory examined the potential involvement of brain MCs in food allergy, an atopic disease with increased peripheral MC activity in response to ingested allergens and often associated with behavioral or mood changes [35,42,43,44]. Using a mouse model of subclinical cow’s milk allergy (CMA) without immediate overt reactions, we demonstrated that the number and degranulation of brain MCs increased in whey protein-sensitized male mice after an acute allergen challenge, but not in female mice [45]. The MC accumulation in sensitized males was accompanied by moderately but significantly elevated allergen-specific serum IgE levels and reduced burrowing behavior. Our observations suggested that allergy-induced peripheral inflammation could result in the activation of central MCs and behavioral changes, at least in male mice. However, how intracranial MCs are stimulated in sensitized mice, how degranulated MCs influence brain function, and whether meningeal MCs are also invloved remains to be elucidated. Furthermore, whether repeated allergen exposure, which likely occurs in allergen-sensitized individuals with subclinical symptoms, results in chronic brain MC activation is yet to be investigated.

We hypothesized that MCs recruited to the brain and meninges in sensitized individuals could be activated by offending allergens via the IgE/FcεRI mechanism and increase BBB permeability to promote neuroinflammation by releasing histamine and other inflammatory factors. In addition, we postulated that intracranial MC accumulation and activation could persist with repeated allergen exposure. To test our hypothesis, we used our subclinical CMA mouse model, in which male C57BL6/J mice were sensitized to a bovine whey allergen, β-lactoglobulin (BLG; Bos d 5). After establishing hypersensitivity, the mice were placed on a whey-containing diet for two weeks for repeated allergen exposure. The open-field, elevated zero maze, and tail suspension tests were performed to assess overall activity and affective behavior. Infiltration and activation of MCs in the CNS, the integrity of the BBB, and the levels of histamine and MC proteases were examined in the sensitized mice as MC-associated cellular and molecular phenotypes suggestive of neuroinflammation. Furthermore, changes in the histamine H3 receptor (H3R) levels were also evaluated as a possible consequence of intracranial MC dysregulation affecting the central histaminergic system [46].

## 2. Materials and Methods

### 2.1. Animals and BLG Sensitization

**Figure 1:**
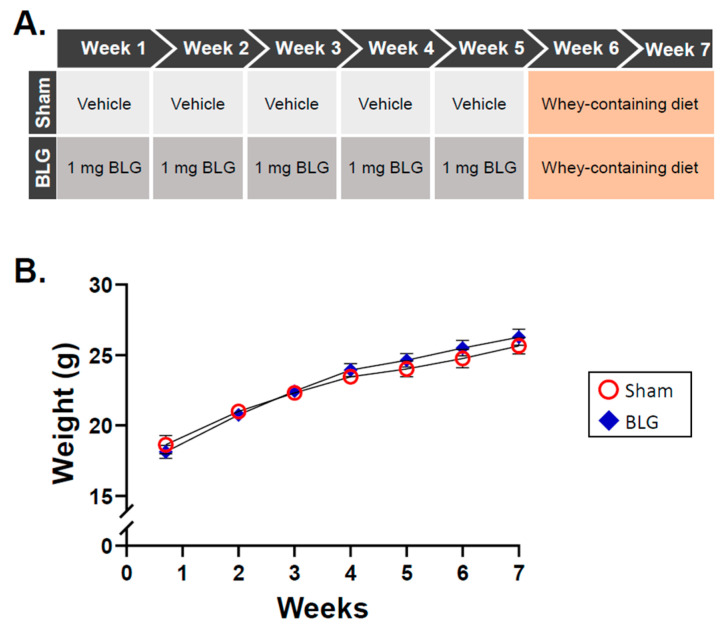
Experimental details and weight changes of the mice during the allergen sensitization and consumption procedures. (**A**) The mice were randomly assigned to the sham group or the BLG treatment group upon arrival (week 0) and subjected to a 5-week sensitization regimen followed by a 2-week allergen exposure period. At each weekly sensitization, the mice received an intragastric administration of either 200 µL of the vehicle containing 10 μg cholera toxin (CT) or 1 mg BLG in the vehicle. Starting at week 6, all the mice were placed on a whey-containing diet for 2 weeks. Behavioral testing was carried out one day before sacrifice in week 7. (**B**) The growth of the sham and BLG-sensitized mice was monitored by measuring their body weight weekly during the course of the experiment. Red open circles and blue diamonds represent the group averages ± SEM for the sham mice and the BLG mice, respectively, at each timepoint (*n* = 10 per group).

Three-week-old male C57BL/6J mice were purchased from Jackson Laboratories (Bar Harbor, ME, USA). The mice were housed five per cage under a 12 h light / 12 h dark cycle and had ad libitum access to water and a whey-free diet (Teklad 2018, Envigo Corporation, Indianapolis, IN, USA). BLG sensitization was carried out according to our prior work [47,48] with modifications. Once the mice reached 4 weeks of age, they were randomly assigned to the sham group or the BLG sensitization group and subjected to a weekly sensitization schedule for 5 weeks (Figure 1A). Both treatment groups were fasted for 2 h before and after sensitization to ensure the absence of ingesta. The BLG-sensitized group was gavaged with 200 µL sodium carbonate/bicarbonate buffer (pH 9.0) containing 10 µg cholera toxin (CT; List Biologicals, Campbell, CA, USA) as an adjuvant and 1 mg of purified BLG (Millipore Sigma, Burlington, MA, USA). The sham mice received only the vehicle (10 µg CT in 200 µL sodium carbonate/bicarbonate buffer). On week 6, both the sham and BLG-sensitized mice were placed on a rodent diet containing 0.3% whey protein (Teklad 8640, Envigo) and allowed to freely feed upon the diet for additional 2 weeks (Figure 1A). The mice were weighed weekly to ensure their growth was not affected by allergen sensitization or aversion to the whey diet (Figure 1B), and signs of allergic symptoms were monitored closely. All the procedures in this study were in compliance with the Guide for the Care and Use of Laboratory Animals of the National Institutes of Health and approved by the University of North Dakota Institutional Animal Care and Use Committee.

### 2.2. Blood and Tissue Collection

One day before the first sensitization in week 1 and at week 5, blood was collected from the tail veins of the sham and BLG mice in EDTA-coated tubes (Sarstedt, Inc., Newton, NC, USA). Plasma samples were isolated and stored at −80 °C until use. One day after behavioral testing during week 7, the animals were sacrificed by CO_2_ asphyxiation. Atrial blood was collected into uncoated microfuge tubes and EDTA-coated tubes, and the serum and plasma were isolated and stored at −80 °C until use. The mice were perfused with the phosphate-buffered saline (PBS; pH 7.4) to clear the remaining blood. The brain was removed from the remaining skull and bisected sagittally. The left hemisphere was immersion-fixed in 4% paraformaldehyde (PFA) for 48 h at 4 °C. The right hemisphere was microdissected into nine regions (olfactory bulb, frontal cortex, striatum, parietotemporal cortex, hippocampus, thalamus/hypothalamus, midbrain, cerebellum, and brainstem) as described previously [47] and stored frozen at −80 °C until use. The dural tissues used for immunochemical staining of FcεRI and IgE were collected from another set of sham and BLG-sensitized mice that had received an additional sensitization dose after 2 weeks of the whey-containing diet and continued the diet for additional 2 weeks. The calvarium, to which the dura mater of the meninges was attached, was carefully separated from the base of the skull and fixed in 4% PFA for 24 h at 4 °C. Fixed dural tissue was carefully peeled from the skull, rinsed in PBS, whole-mounted on a subbed glass slide, and dried overnight.

### 2.3. Enzyme-Linked Immunosorbent Assays (ELISAs)

#### 2.3.1. BLG-Specific IgE and IgG1

Isotype-specific detection of immunoglobulins (Igs) was carried out according to the protocol by Germundson and Nagamoto-Combs [49] with modifications. Eight-well RIA strips (Corning, Inc., Corning, NY, USA) were coated with 2 μg/mL BLG in a sodium carbonate/bicarbonate buffer (pH 9.5) overnight at 4 °C. The wells were washed and blocked in the PBS containing 0.5% bovine serum albumin (BSA). The serum samples were diluted to 1:40 and incubated with protein G-coated plates (Thermo Fisher, Waltham, MA, USA) for 1 h at 37 °C to adsorb total IgG, and the resulting supernatant was subsequently added to each well. Allergen-specific IgE was detected using secondary anti-mouse IgE and avidin HRP (eBioscience, San Diego, CA, USA) [49]. The substrate reaction was terminated with 2N sulfuric acid, and the plates were immediately read at 450 nm with a reference wavelength of 550 nm on an ELx800 Universal Microplate Reader (BioTek Instruments, Winooski, VT, USA).

#### 2.3.2. Histamine and Mast Cell Protease-1 (MCPT-1)

The serum samples collected at the time of sacrifice were used at a 1:10 dilution to quantify histamine and MCPT-1 as indicators of systemic and brain MC activities. For histamine detection, a competitive histamine ELISA kit was used (Enzo Life Sciences, Inc., Farmingdale, NY, USA). The diluted terminal plasma samples (1:10) and 50 µg/mL of the brain lysates were used to determine histamine levels according to the manufacturer’s instructions. MCPT-1 was detected using a Mouse MCPT-1 Uncoated ELISA Kit according to the manufacturer’s instructions (Thermo Fisher Scientific, Waltham, MA, USA).

#### 2.3.3. Serum BLG

The concentration of BLG present in the blood and brain was quantified using a Bovine Beta-Lactoglobulin (bLg) DIY ELISA Kit (MyBioSource, Inc., San Diego, CA, USA) to determine increases in transepithelial transport of the allergen through impaired intestinal barriers. The diluted terminal serum samples (1:10 dilution) or 50 µg/mL of the brain lysates were used to perform the assays according to the manufacturer’s instructions.

### 2.4. Behavior Analysis

#### 2.4.1. Open-Field Test (OFT)

The OFT was used to evaluate overall mobility and anxiety-like behavior as described previously [48]. Briefly, the mice were individually placed in an open-field apparatus with opaque walls (San Diego Instruments, San Diego, CA, USA) and allowed to acclimate for 30 s. The activity of each mouse was then video-recorded for 10 min. Changes in mobility (time mobile, distance traveled, number of immobile episodes) and anxiety-like behaviors (frequency and duration of visits to the center zone of the apparatus) were analyzed using the ANY-maze software (Stoelting Co., Wood Dale, IL, USA). Behavior was analyzed for the first (0–5 min) and the second (6–10 min) halves separately to assess potential time-dependent differences during the 10 min recording session [48,50,51,52]. The OFT and all other behavioral test apparatuses were thoroughly cleaned between animal testing with Process NPD (STERIS, Mentor, OH, USA).

#### 2.4.2. Elevated Zero Maze (EZM)

An EZM apparatus (Stoelting Co.) was used to measure anxiety-like behavior [48]. The mice were individually placed in one of the walled sections of the circular maze and allowed to explore the apparatus freely for 10 min while being video-recorded. The time spent in the open zones, the number of entries into the open zones, and the average duration of visits to the open zones were analyzed using the ANY-maze software and validated manually by a blinded observer. One BLG-sensitized mouse was removed from the final analysis due to falling. Avoidance of the open zones was considered anxiety-like behavior.

#### 2.4.3. Tail Suspension Test (TST)

Depression-like behavior was evaluated with the TST as described previously [47,48]. Briefly, the mice were suspended by the tail from a horizontal bar with a piece of laboratory tape approximately 30 cm above the base of the bar support. A piece of plastic tubing was used to prevent the mice from climbing their tails during the testing period [53]. Their movements were video-recorded for 6 min, and the frequency and length of immobility were compared between the treatment groups as indications of depression-like behavior [54].

### 2.5. Western Blotting

Protein extracts from the microdissected brain regions were prepared as described previously [47] with modifications. Soluble proteins were first isolated in the PBS supplemented with Phosphatase Inhibitor Cocktail II (Thermo Fisher Scientific). The remaining insoluble pellets were washed with the PBS and resuspended in the RIPA buffer (20 mM Tris, 150 mM sodium chloride, 1 mM sodium orthovanadate, 10 mM sodium fluoride, 1 mM EDTA, 1 mM EGTA, 0.2 mM phenylmethanesulfonyl fluoride, 1% Triton X-100, 0.1% SDS, and 0.5% deoxycholate; pH 7.4). The total protein concentrations were determined using the Bradford assay [54]. The protein samples (20 μg) were resolved on 10% or 15% SDS–polyacrylamide gels and transferred onto Immobilon-P PVDF membranes (MilliporeSigma). The membranes were incubated in an Intercept^®^ (TBS) Blocking Buffer (LI-COR, Lincoln, NE, USA) for 1 h at room temperature and with primary antibodies (Table 1) overnight at 4 °C. Appropriate IRDye^®^-conjugated secondary antibodies (1:10,000; LI-COR) were used for simultaneous visualization of the target and reference proteins, glyceraldehyde-3-phosphate dehydrogenase (GAPDH) or α-tubulin, using an Odyssey-CLx Infrared Imaging System (LI-COR). The fluorescence signals were quantitated using Empiria Studio 2.0 (LI-COR), and the amounts of the target proteins were normalized to the reference protein in each sample.

### 2.6. Immunohistochemical Staining

#### 2.6.1. Brain

Brain tissue samples were prepared for histological analysis as previously described [55]. Briefly, the fixed left-brain hemispheres were embedded in gelatin, frozen-sectioned at 40 µm, and stored in a cryoprotectant (0.1 M phosphate buffer (pH 7.4) containing 30% (*w*/*v*) sucrose, 30% (*v*/*v*) ethylene glycol) at −20 °C until use. Following treatment with 0.3% (*v*/*v*) hydrogen peroxide and blocking with 0.5% (*w*/*v*) BSA, 0.1% (*v*/*v*) Triton X-100, and 10% normal goat serum at room temperature, the brain sections were incubated with the rabbit anti-mouse glial fibrillary acidic protein antibody (GFAP; 1:1000; Cell Signaling Technologies, Danvers, MA, USA) or the anti-mouse IgG antibody (1:500; eBioscience, Thermo scientific) overnight at 4 °C. Immunoreactivity was visualized using a Vectastain Elite ABC-HRP Kit with an appropriate species-specific biotinylated secondary antibody (1:2000) and a VIP Peroxidase Substrate Kit (Vector Laboratories, Burlingame, CA, USA) as the chromogen. The brain sections were mounted on gelatin-subbed glass slides, dehydrated through ethanol gradients, and cleared with Histo-Clear II (National Diagnostics, Atlanta, GA, USA) before being coverslipped.

#### 2.6.2. Dura Mater

The air-dried, whole-mounted dural tissues were briefly rehydrated and incubated in a Tris–EDTA buffer containing 0.01% Tween (pH 9.0) overnight at 37 °C for antigen retrieval. The rabbit anti-mouse FcεRI (Invitrogen, Thermo Scientific) and goat anti-mouse IgE (Novus, Centennial, CO, USA) antibodies were used at 1:200 dilution. Immunoreactivity was visualized as described above.

### 2.7. Black Gold II Staining

The brain sections were mounted on gelatin-coated slides and allowed to dry overnight. Following the manufacturer’s instructions, the sections were incubated in a preheated Black Gold II staining solution (Biosensis, Thebarton, South Australia, Australia) at 60 °C for 20 min with gentle agitation. The slides were then coverslipped as described above.

### 2.8. MC Staining and Quantification

The brain or meningeal tissues mounted on gelatin-coated slides were metachromatically stained with acidic toluidine blue by incubating the slides in a freshly prepared toluidine blue solution (1% toluidine blue in 70% ethanol diluted 1:10 in acidic 1% NaCl (pH 1.9)) for 2 h (brain) or 1 h (dural tissues). The slides were washed in water, dehydrated, and cleared before being coverslipped. MC numbers in the brain sections were quantified as detailed in our previous work [45]. To quantify the meningeal MCs, a blinded experimenter counted the total number of granulated and degranulated MCs per 40× field for a total of 20 fields using an Olympus BX63 brightfield microscope (Waltham, MA, USA). The mean of the 20 fields per section was calculated as the average MCs per field. The sinus regions of the dura were excluded from MC quantification since it was difficult to distinguish individual MCs in these regions due to the extensive overlap of MCs.

### 2.9. Densitometric Analysis of Histological Staining

The histological slides were scanned using a Hamamatsu NanoZoomer 2.0HT Brightfield + Fluorescence Slide Scanning System (Hamamatsu Photonics, Bridgewater, NJ, USA). The exposure settings were kept consistent for comparison. The image files (.ndp) were imported into the QuPath v0.3.0 image analysis software [56]. To determine the optical density (OD), the backgrounds of the slides were normalized for each treatment group (staining vectors), and the region of interest (ROI) was defined using a shape tool in the QuPath software. The average OD of the staining in the ROI per 2 μm was determined by QuPath from three serial sections and expressed as the relative OD for the ROI.

### 2.10. Statistical Analysis

All the statistical analyses were performed using the GraphPad Prism v9.0 software (GraphPad Software, Inc., La Jolla, CA, USA). The differences between the sham group and the BLG-sensitized group were compared using Student’s *t*-test or multiple uncorrected *t*-tests. The ROUT method (Q = 1%) was used to identify outliers in the sham group or the BLG group when appropriate, and the values were removed from the final results. A *p*-value less than 0.05 (*p* < 0.05) was considered statistically significant.

## 3. Results

### 3.1. Repeated Allergen Consumption Resulted in Asymptomatic Hypersensitivity with Decreased Mobility and Depression-like Behavior in the BLG-Sensitized Mice

**Figure 2:**
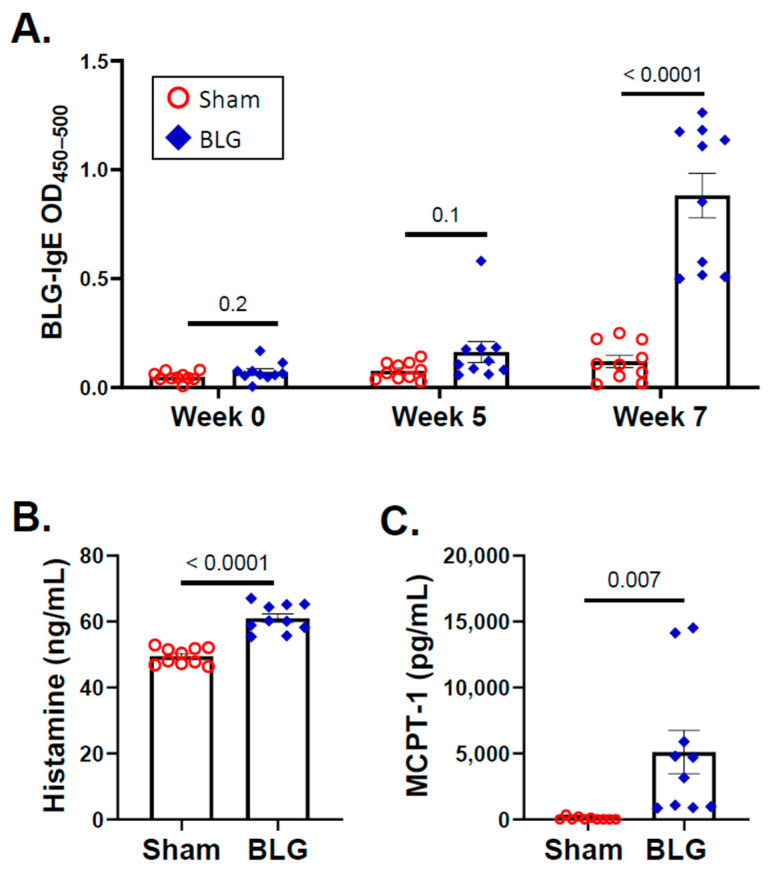
Quantification of the BLG-specific IgE, histamine, and mast cell protease-1 (MCPT-1) using ELISA. The blood samples collected from the sham and BLG-sensitized mice at weeks 0, 5, and 7 were used to detect the plasma levels of BLG-specific IgE (**A**). The values indicate spectrophotometric readouts at 450 nm after subtracting the reference readouts at 550 nm and are expressed as optical density (OD). Histamine (**B**) and MCPT-1 (**C**) were also quantified from the plasma and serum samples of the terminal blood, respectively, using commercial ELISA kits with respective standards. Each bar indicates the group average ± SEM (*n* = 10 per group). Red open circles and blue diamonds represent individual values for the sham and BLG mice, respectively. The numbers between two bars indicate the *p*-values. Statistical significance (*p* < 0.05) was determined with multiple uncorrected *t*-tests (BLG-specific IgE) or unpaired Student’s *t*-tests (histamine and MCPT-1).

As we previously reported [47,48], no differences in growth were observed between the sham and BLG-sensitized animals during the 5-week sensitization period, the transition to the whey-containing diet, or the subsequent two weeks of allergen consumption (Figure 1B). Moreover, the sensitized mice did not display any observable physical symptoms of allergic reactions throughout the experiment. However, the levels of BLG-specific IgE in the sensitized group were elevated approximately twofold by week 5 (sham: 0.08 ± 0.01; BLG: 0.16 ± 0.05) and further increased to 7.5-fold by the end of week 7 (sham: 0.1 ± 0.03; BLG: 0.9 ± 0.1; *p* < 0.0001) (Figure 2A). Moreover, the levels of both histamine (sham: 49 ± 0.8 pg/mL; BLG: 61 ± 1 pg/mL; *p* < 0.0001) and MCPT-1 (sham: 72 ± 41 pg/mL; BLG: 5102 ± 1647 pg/mL; *p* = 0.007) were significantly greater in the plasma and serum, respectively, than in the sham group (Figure 2B,C), suggesting that MCs had been activated. Together, these results validated our previous findings, demonstrating that BLG sensitization induced the development of asymptomatic hypersensitivity in the male C57BL/6J mice. In addition, allergen-specific IgE levels continued to increase during the 2-week allergen exposure period, indicating that repeated consumption of whey further heightened the humoral immune responses of the BLG-sensitized mice.

**Figure 3:**
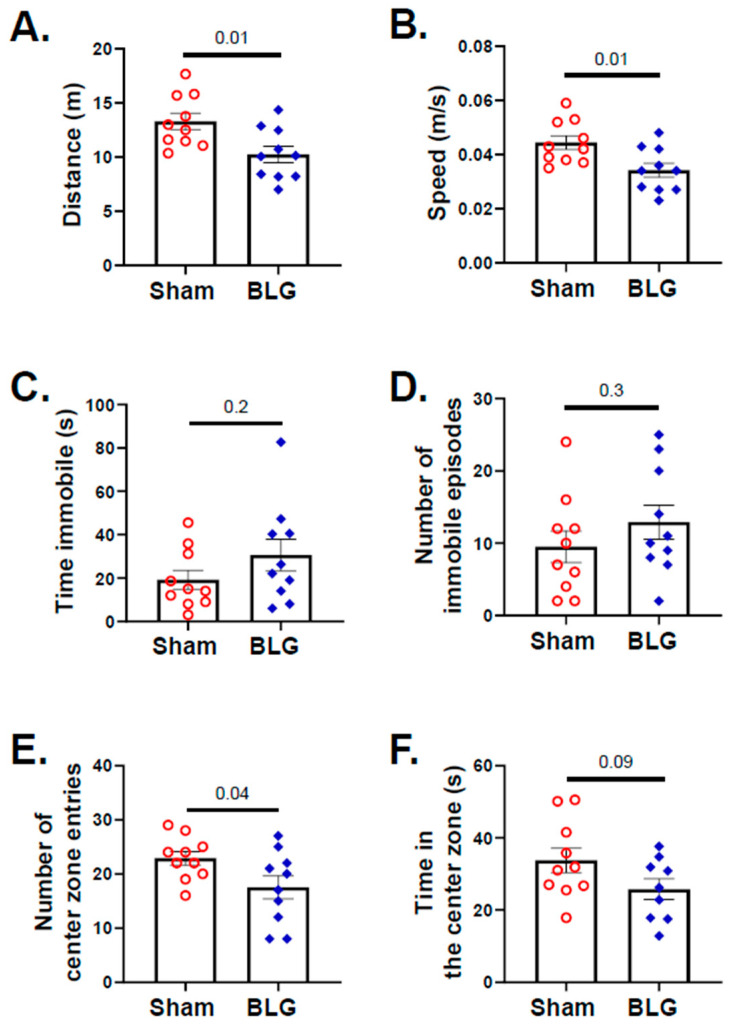
Assessment of the overall activity and anxiety-like behavior with the open-field test (OFT). All the mice were subjected to the OFT during week 7, and their activity was recorded for 10 min. The distance (**A**), speed (**B**), total time immobile (**C**), number of immobile episodes (**D**), number of center zone entries (**E**), and time spent in the center zone (**F**) were analyzed using the ANY-maze software from the recording. Each bar indicates the group average ± SEM (*n* = 10 per group). Red open circles and blue diamonds represent individual values for the sham and BLG mice, respectively. The numbers between the two bars indicate the *p*-values. Statistical significance (*p* < 0.05) was determined using an unpaired Student’s *t*-test.

During the second week of the allergen exposure period in week 7, the mice were subjected to a series of behavioral tests. When various locomotor parameters, such as speed, distance traveled, immobile episodes, and the frequency and time in the center zone, were quantified for the entire test duration with the OFT, no significant differences were observed between the sham group and the BLG-sensitized group. However, when these activities were plotted as a function of time (Figure S1), it became apparent that the BLG-sensitized mice traveled a significantly smaller distance (sham: 13 m ± 0.8; BLG: 10 m ± 0.8; *p* = 0.01) and moved at lower speeds (0.04 m/s ± 0.003; BLG: 0.03 m/s ± 0.003; *p* = 0.01) than the sham mice during the second half (6–10 min) of the testing period (Figure 3A,B). Such time-dependent differences between the groups were also present with the duration and number of immobile episodes (Figure S1C,D) but did not reach statistical significance (Figure 3C,D). The number of center-zone entries made by the BLG-sensitized mice was also significantly lower than that by the sham mice during the second half of the test (Figure 3E: sham: 22 ± 1; BLG: 17 ± 2; *p* = 0.04), while the total time they spent in the center zone was not statistically different from the sham mice (Figure 3F).

**Figure 4:**
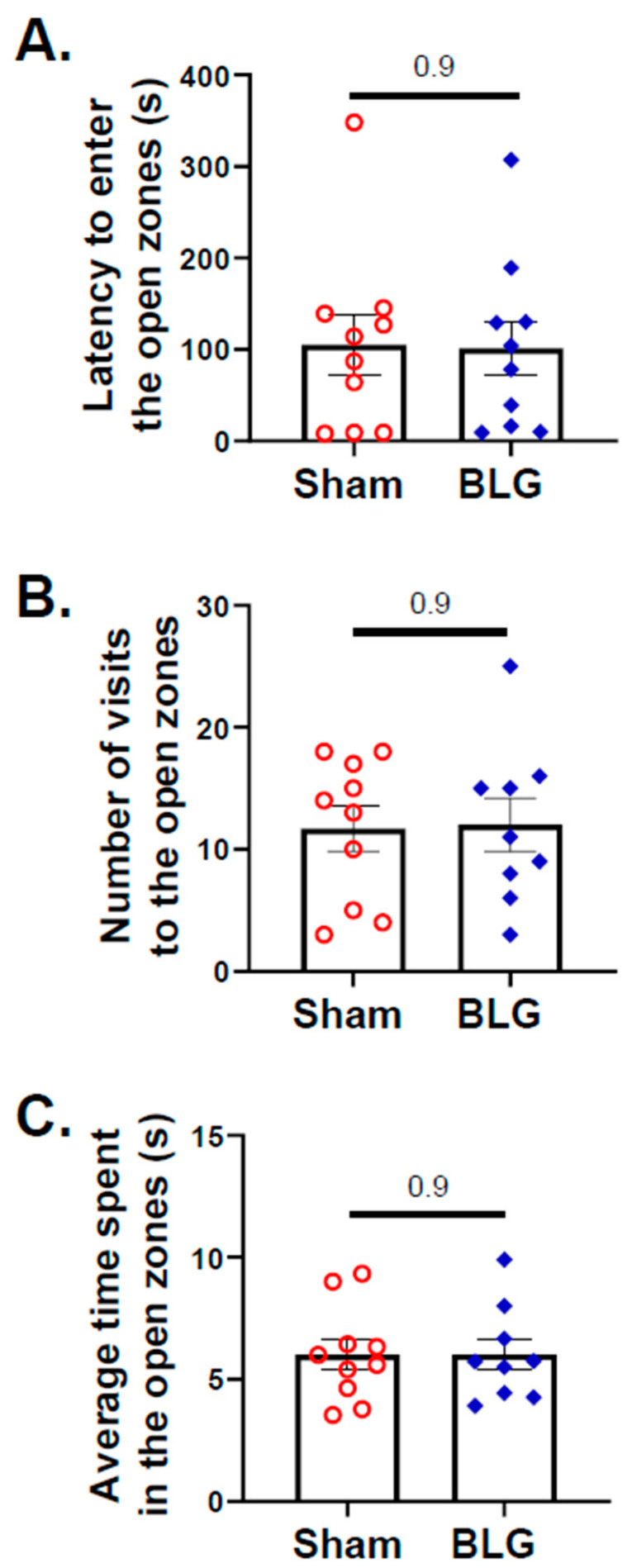
Assessment of height-induced anxiety-like behavior with the elevated zero maze (EZM). The activity of mice on the EZM was recorded for 10 min. Latency to enter the open zones (**A**), the number of visit to the open zones (**B**), and the average time spent in the open zones (**C**) were analyzed from the recording using ANY-maze software. Each bar indicates the group average ± SEM (*n* = 9–10 per group). Red open circles and blue diamonds represent individual values for sham and BLG mice, respectively. The numbers between two bars indicate the *p*-values. Statistical significance (*p* < 0.05) was determined by an unpaired Student’s *t*-test.

**Figure 5:**
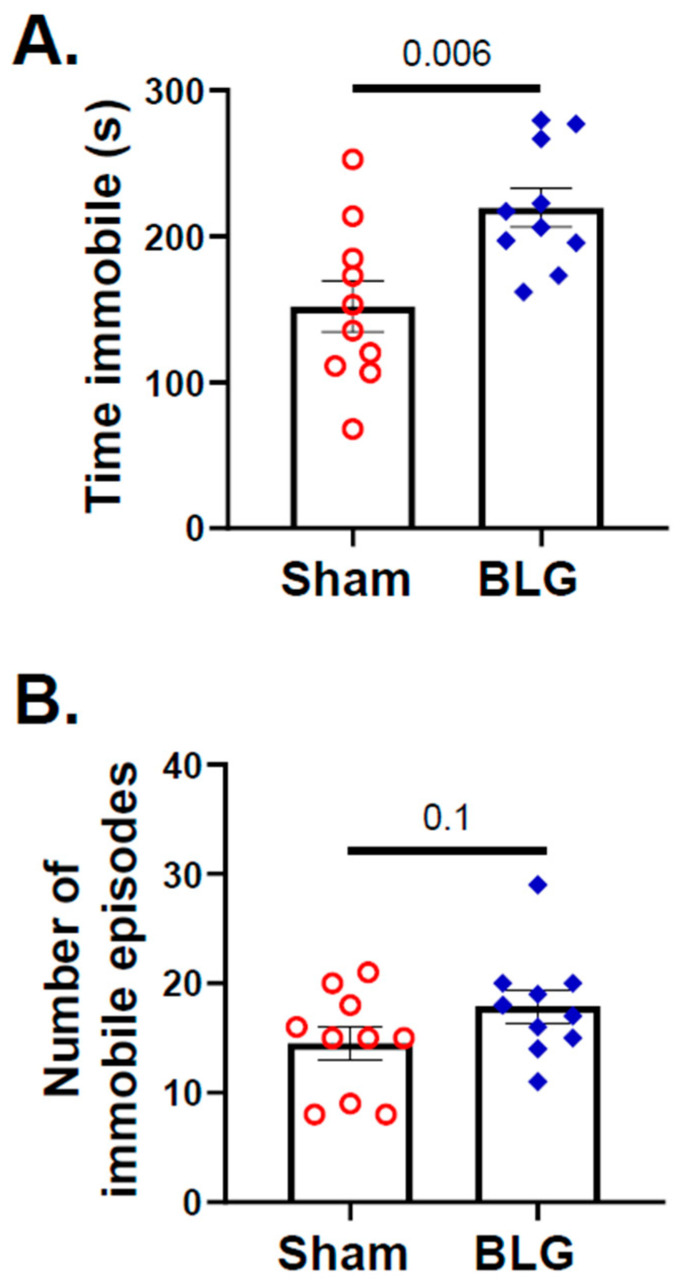
Assessment of immobility as depression-like behavior with the tail suspension test (TST). The activity of sham and BLG-sensitized mice in the suspension during the TST was recorded for 6 min. The time spent immobile (**A**) and the number of immobile episodes (**B**) were quantified from the video recording using the ANY-maze software. Each bar indicates the group average ± SEM (*n* = 10 per group). Red open circles and blue diamonds represent individual values for the sham and BLG mice, respectively. The numbers between the two bars indicate the *p*-values. Statistical significance (*p* < 0.05) was determined using Student’s *t*-test.

In contrast to the indication of anxiety-like behavior in the BLG-sensitized mice during the second half of the OFT, no differences in the entries to the open zones between the sham group and the sensitized group were detected by the EZM (Figure 4A–C). However, indications of depression-like behavior were present when the mice were subjected to the TST, with the BLG-sensitized mice spending significantly greater immobile time (sham: 151 ± 18 s; BLG: 220 ± 13 s; *p* = 0.006) compared to the sham mice (Figure 5A). The frequencies of immobile episodes exhibited by the sensitized mice also showed a higher trend, but the difference was not statistically significant (Figure 5B). These results indicated that the behavior of the BLG-sensitized mice was affected after the repeated allergen exposure. The time-dependent decline in their movement might have influenced their exploratory activity in the OFT and effort to elude their vulnerable position during the TST.

### 3.2. MCs Accumulated in the Brain and Meninges of the BLG-Sensitized Mice and Displayed Activated Morphology

**Figure 6:**
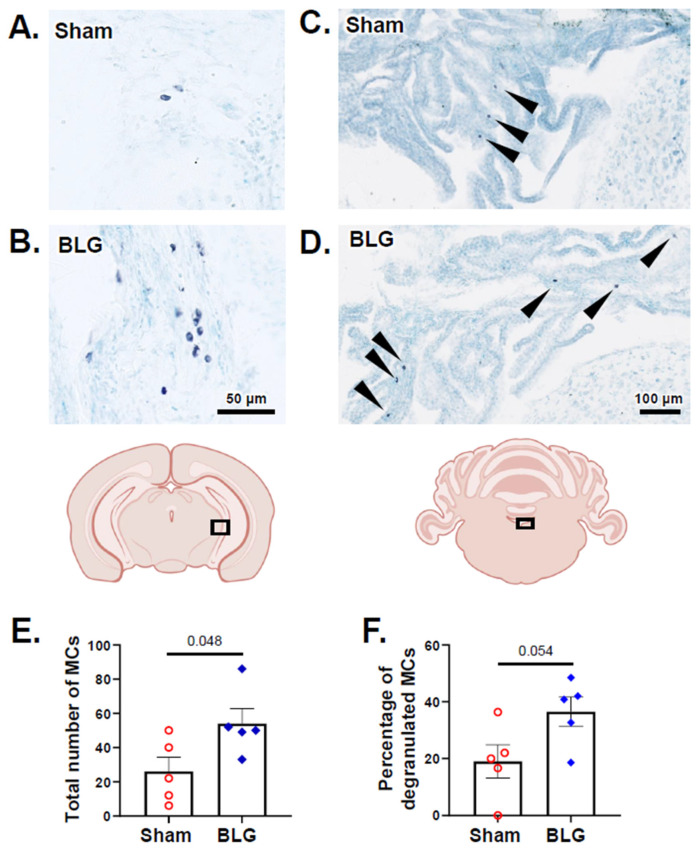
Histological detection and quantification of brain MCs identified with acidic toluidine blue staining. Metachromatically stained MCs in the brain sections from the sham (**A**,**C**) and BLG-sensitized (**B**,**D**) mice were identified using light microscopy. The MCs located in the region between the caudal thalamus/midbrain and the medial hippocampus (**A**,**B**) and the choroid plexus in the fourth ventricle (arrowheads in (**C**,**D**)) were photographed using 40× and 20× objectives, respectively. Scale bars: 50 µm (for (**A**,**B**)); 100 µm (for (**C**,**D**)). From the toluidine blue-stained sham and BLG-sensitized mouse brain sections, the number of total MCs (**E**) and the percentage of degranulated MCs (**F**) were quantified. Each bar indicates the group average ± SEM (*n* = 5 per group). Red open circles and blue diamonds represent individual values for the sham and BLG mice, respectively. The numbers between the two bars indicate the *p*-values. Statistical significance (*p* < 0.05) was determined using Student’s *t*-test.

**Figure 7:**
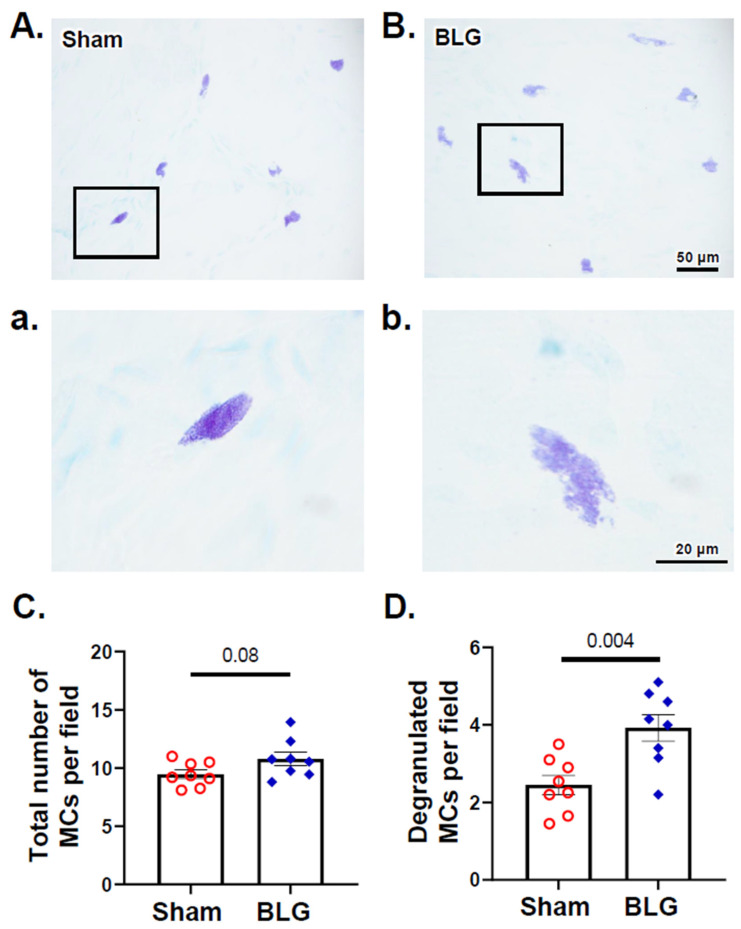
Histological detection and quantification of dural MCs identified with acidic toluidine blue staining. Metachromatically stained MCs in the dura mater from the sham (**A**) and BLG-sensitized (**B**) mice were identified using light microscopy and photographed using a 40× objective. Higher magnification images of MCs (**a**,**b**) were captured using a 100× objective. Scale bars: 50 µm (for (**A**,**B**)) and 20 µm (for (**a**,**b**)). The number of total MCs (**C**) and the percentage of degranulated MCs (**D**) were quantified from the toluidine blue-stained dural tissues collected from the sham and BLG-sensitized mice. Each bar indicates the group average ± SEM (*n* = 8 per group). Red open circles and blue diamonds represent individual values for the sham and BLG mice, respectively. The numbers between the two bars indicate the *p*-values. Statistical significance (*p* < 0.05) was determined using Student’s *t*-test.

After 2 weeks of repeated allergen consumption, we quantified metachromatically stained MCs in the brains of the sham and BLG-sensitized mice. MCs were found sparsely in the brain parenchyma but more frequently in the choroid plexus and leptomeninges for both groups (Figure 6A–D). There were significantly greater numbers of brain MCs in the sensitized mice (Figure 6E; sham: 26 ± 18; BLG: 54 ± 19; *p* = 0.048), and the difference from the sham mice was particularly evident in the leptomeninges located between the thalamus/midbrain and the medial hippocampus (Figure 6A,B). Many of the MCs were in a degranulated state, although the quantitative comparison with the sham mice did not reach statistical significance (Figure 6F; *p* = 0.054). In the dura mater, MCs were numerous in both the sham mice and the BLG-sensitized mice (Figure 7A,B). The number of total MCs per high-powered field trended greater in the BLG-sensitized mice than in the sham mice, although the difference was not statistically significant (*p* = 0.08) (Figure 7C). In contrast, the number of degranulated MCs was significantly higher in the BLG-sensitized mice (sham: 2.5 ± 0.3; BLG: 3.9 ± 0.3; *p* = 0.004) (Figure 7D), suggesting that dural MCs had been activated in this group.

**Figure 8:**
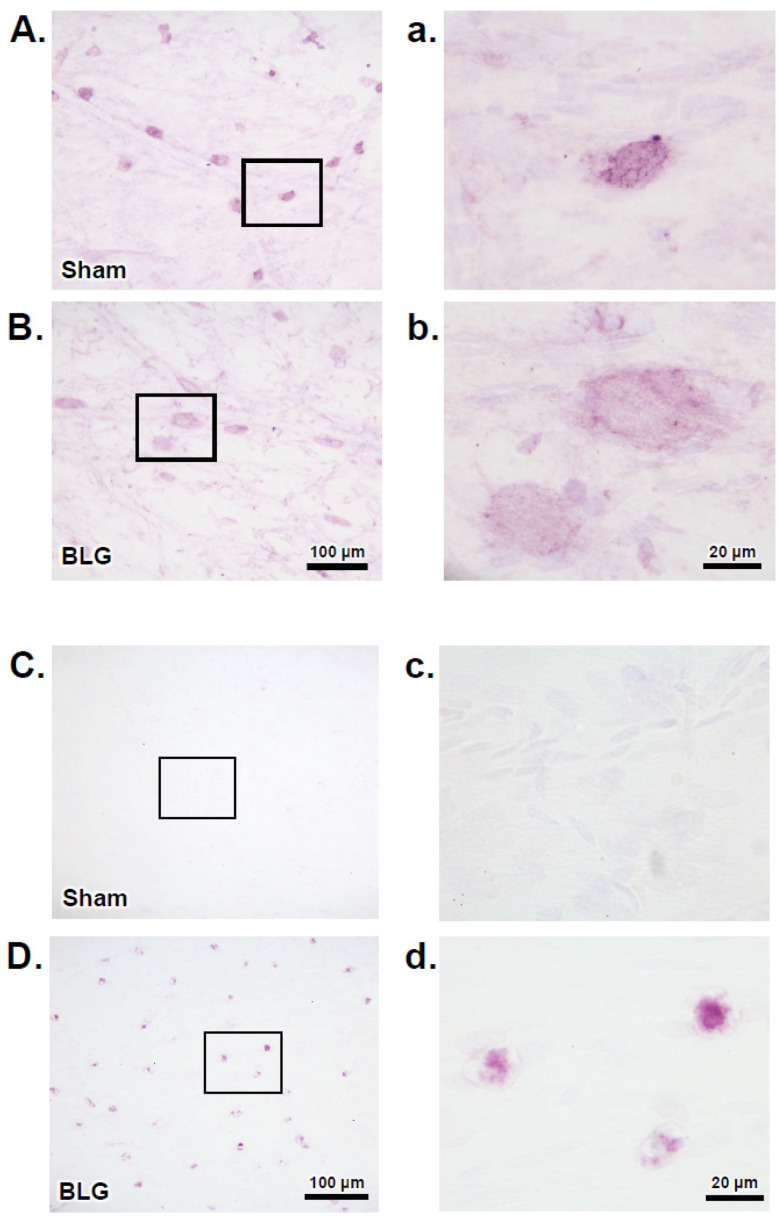
Immunohistochemical detection of FcεRI and IgE in dural MCs. Dural tissues were collected from the sham and BLG-sensitized mice with prolonged whey-containing diet and stained using antibodies against FcεRI (**A**,**B**) and IgE (**C**,**D**). The rectangles in panels A–D denote the areas where the corresponding high-magnification photomicrographs in panels a-d were taken. The immunoreactive cells were photographed with 40× (**A**–**D**) and 100× objectives (**a**–**d**). Scale bars: 100 µm (for panels (**A**–**D**)); 20 µm (for panels (**a**–**d**)).

The apparent difference in the number of degranulated MCs in the dura led us to question whether the meningeal MCs of the sensitized mice had undergone IgE/FcεRI-mediated degranulation. To address this question, we first determined whether these MCs expressed FcεRI, and if so, whether the receptors were primed with IgE. Immunohistochemical detection of FcεRI indicated that MCs of both the sham and BLG-sensitized mouse brains expressed FcεRI (Figure 8A,B). However, immunoreactivity was more robust in the sham mice. In addition to the difference in staining intensity, the immunoreactive cells of the two groups were morphologically distinct. The majority of FcεRI-positive MCs of the sham mice were smaller, with a more defined ovoid cell shape (Figure 8a), while some of the MCs of the BLG-sensitized mice were noticeably larger, and the staining was more diffuse, with a spread-out appearance (Figure 8b). Importantly, when the dural tissues were immunostained for IgE, numerous immunopositive cells were found in the BLG-sensitized mice, whereas no positively stained cells were present in the sham mice (Figure 8C,D). Together, these results indicated that MCs were likely recruited to the CNS during sensitization, and their number and degranulation remained elevated with prolonged allergen exposure. Furthermore, our observations suggested that intracranial MCs were equipped to become activated via the IgE/FcεRI mechanism.

### 3.3. Intracranial MCs Are Likely Activated by Circulating Allergens in the CMA Mice during the Repeated Allergen Exposure

**Figure 9:**
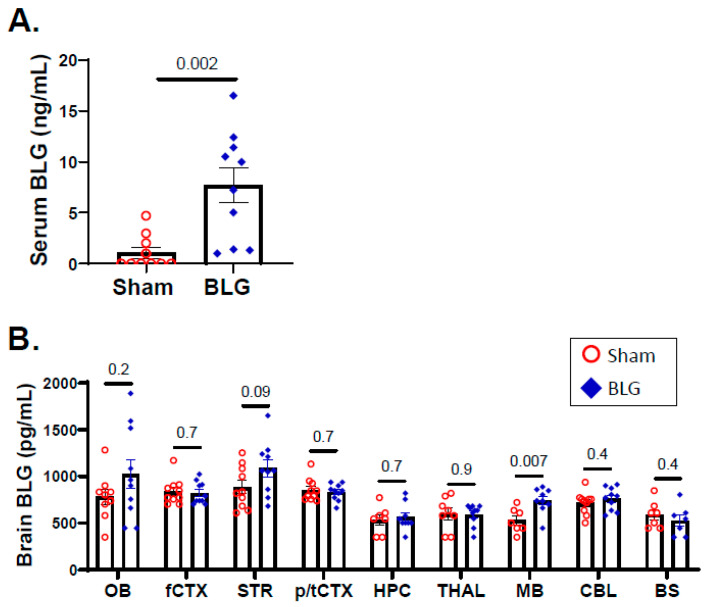
Quantitation of BLG levels in the sera and the brain. The amounts of BLG present in the sera (**A**) and brain lysates (**B**) from the sham and BLG-sensitized mice were quantified using a quantitative ELISA system for bovine BLG. Each bar indicates the group average ± SEM (*n* = 10 per group). Red open circles and blue diamonds represent individual values for the sham and BLG mice, respectively. The numbers between the two bars indicate the *p*-values. Statistical significance (*p* < 0.05) was determined using a Student’s *t*-test (serum BLG) or multiple uncorrected *t*-tests (brain-region specific BLG). OB: olfactory bulb; fCTX: frontal cortex; STR: striatum; p/tCTX: parietotemporal cortex; HPC: hippocampus; THAL: thalamic/hypothalamic region; MB: midbrain region; CBL: cerebellum; BS: brainstem.

Our observations thus far have suggested the involvement of IgE/FcεRI-induced intracranial MC degranulation. Since the activation of FcεRI signaling is initiated by antigen binding to the FcεRI-associated IgE [57], we next measured the amount of BLG in the blood and brain tissues to determine whether the allergen would have access to intracranial MCs and trigger their activation. As suspected, the amount of BLG was significantly elevated in the sera of sensitized mice after the repeated allergen consumption (sham: 1.1 ± 0.5 ng/mL; BLG: 7.7 ± 1.7 ng/mL; *p* = 0.002) (Figure 9A), suggesting that BLG in the circulation could reach the intracranial MC population through the cerebral vasculature. In support of this notion, the midbrain showed significantly greater levels of BLG in sensitized mice (Figure 9B; sham: 530 ± 49 pg/mL; BLG: 743 ± 45 pg/mL; *p* = 0.007), with trends for increased amounts of the allergen in the olfactory bulb and striatum. However, the allergen was not detected above the sham levels in other brain regions analyzed, indicating that the amount of the CNS-infiltrating allergen is region-dependent. Accordingly, these results also suggested that the activation pattern of MCs, at least within the brain parenchyma, might be distinct at different locations in the brain.

### 3.4. Histamine Levels and H3R Expression Were Regionally Elevated in Brains of the BLG-Sensitized Mice after the Repeated Allergen Consumption

MC degranulation releases histamine, and this event was validated in the periphery by the elevated histamine levels in the sera of the BLG-sensitized mice (Figure 2B). Since we found that MCs had accumulated and been activated in the brains of the CMA mice after repeated allergen exposure, we assessed whether histamine levels were also elevated in the brain. In addition, we quantified H3R immunoreactivity as an indication of central histaminergic system modification. Although we previously demonstrated that H3R expression was elevated in the CMA mice that had been sensitized and acutely challenged with whey proteins [46], this observation required validation with the BLG-sensitized mice after the repeated allergen challenge.

**Figure 10:**
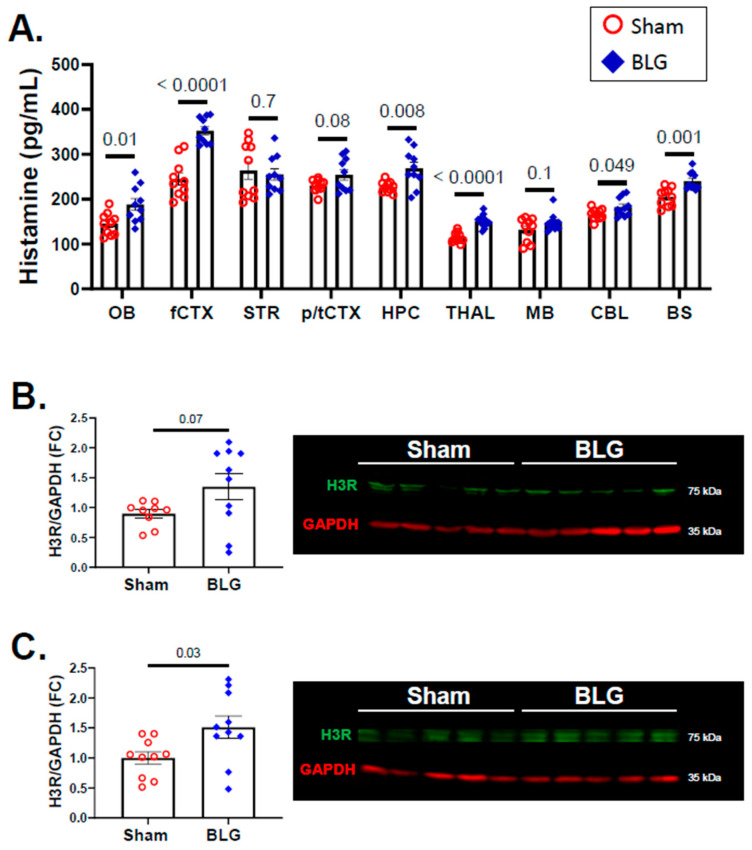
Quantitation of histamine and H3R in the brain. The histamine levels in different regions of the sham and BLG-sensitized mouse brains were quantified using ELISA (**A**). H3R levels from the frontal cortex (**B**) and the parietotemporal cortex (**C**) were quantified using western blotting. The fluorescence signals from the H3R bands (green bands at 65 kDa) were normalized to GAPDH (red bands at 35 kDa) in the corresponding lanes and expressed as fold change (FC) calculated from the average value of the sham mice. Each bar indicates the group average ± SEM (*n* = 10 per group). Red open circles and blue diamonds represent individual values for the sham and BLG mice, respectively. The numbers between the two bars indicate the *p*-values. Statistical significance (*p* < 0.05) was determined using multiple uncorrected *t*-tests (brain region-specific histamine) or Student’s *t*-test (H3R expression). One outlier was removed from the sham group in the H3R western blot analysis by ROUT. OB: olfactory bulb; fCTX: frontal cortex; STR: striatum; p/tCTX: parietotemporal cortex; HPC: hippocampus; THAL: thalamic/hypothalamic region; MB: midbrain region; CBL: cerebellum; BS: brainstem.

When measured using competitive ELISA, elevated histamine levels were detected in various regions of the BLG-sensitized mouse brains (Figure 10A). These regions included the olfactory bulb (sham: 145 ± 8 pg/mL; BLG: 189 ± 13 pg/mL; *p* = 0.01), frontal cortex (sham: 245 ± 13 pg/mL; BLG: 353 ± 9 pg/mL; *p* < 0.0001), hippocampus (sham: 228 ± 4 pg/mL; BLG: 269 ± 13 pg/mL; *p* = 0.008), thalamus (sham: 115 ± 4 pg/mL; BLG: 151 ± 5 pg/mL; *p* < 0.0001), cerebellum (sham: 167 ± 4 pg/mL; BLG: 183 ± 7 pg/mL; *p* = 0.049), and brainstem (sham: 205 ± 6 pg/mL; BLG: 241 ± 6 pg/mL; *p* = 0.001). The striatum, parietotemporal cortex, and midbrain did not show significant differences in histamine contents between the experimental groups, indicating that the changes in histamine levels were regionally distinct.

In addition to the changes in the brain histamine levels, the expression of H3R showed an increasing trend in the frontal cortex of the BLG-sensitized mice, although the difference did not reach statistical significance (*p* = 0.07; Figure 10B). However, there was a 0.5-fold increase in the parietotemporal cortex (*p* = 0.03; Figure 10C) of the BLG-sensitized mice. These findings suggested that the accumulation and activation of brain MCs in the BLG-sensitized mice contributed to the regional histamine elevation and that the increased levels were sustained during prolonged allergen exposure. Furthermore, the altered H3R levels suggested that the altered histamine content might have also dysregulated the function of the central histaminergic system in the cortical regions of the brain.

### 3.5. Activation of Intracranial MCs Was Associated with BBB Impairment and Perivascular Astrogliosis

**Figure 11:**
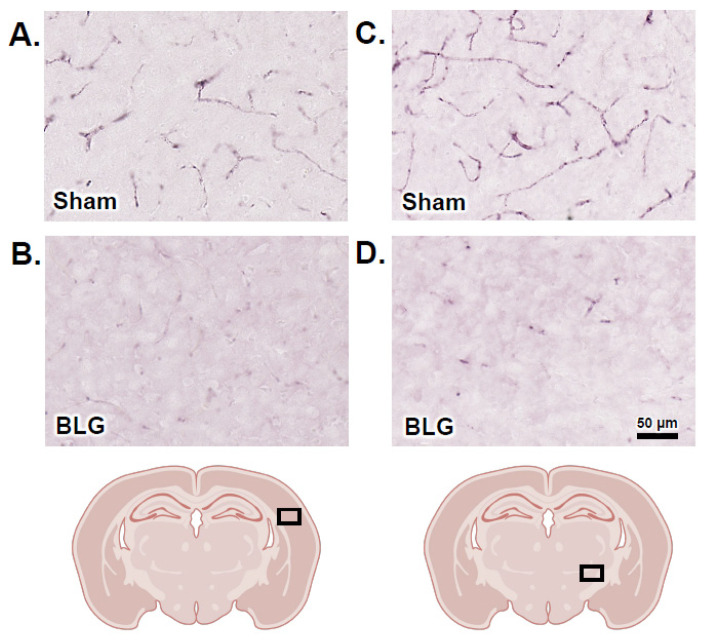
Immunohistochemical staining for the detection of IgG extravasation. Brain sections from the sham (**A**,**C**) and BLG-sensitized (**B**,**D**) mice were subjected to immunohistochemical staining for IgG. Photomicrographs were taken from cortical (**A**,**B**) and thalamic (**C**,**D**) regions with a 40× objective. The rectangles in the brain diagrams indicate the approximate locations where the photomicrographs were taken. Scale bar: 50 µm (for all panels).

Since histamine is a known modulator of vascular permeability [58] and was elevated in the sera and brains of the BLG-sensitized mice (Figure 2B and Figure 10A), we next compared the integrity of cerebral vasculature as a measure of BBB permeability between the two groups of mice. For this purpose, the brain sections were immunostained to detect IgG extravasation into the surrounding brain parenchyma through a compromised BBB. In the sham mouse brains, we found strong IgG immunoreactivity contained within the capillaries and small vessels (Figure 11A,C). In contrast, IgG extravasation was apparent in the BLG-sensitized mice, with diffused staining throughout the brain parenchyma. This observation was particularly consistent in the cortical and thalamic regions (Figure 11B,D), staining the parenchyma darker than it appeared in the sham animals.

**Figure 12:**
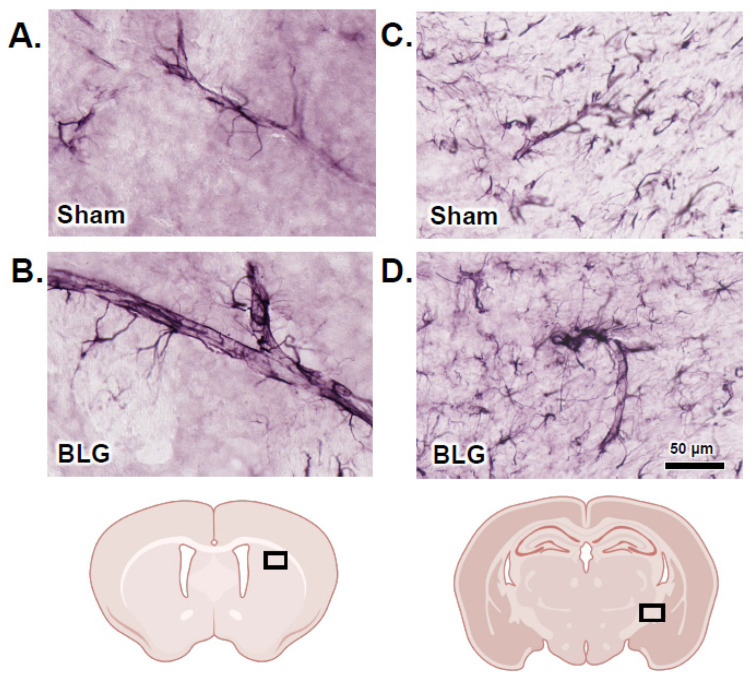
Immunohistochemical staining of glial fibrillary acidic protein (GFAP)-positive perivascular astrocytes. Brain sections from the sham (**A**,**C**) and BLG-sensitized (**B**,**D**) mice were subjected to immunohistochemical staining for GFAP to identify astrocytes. Photomicrographs of GFAP-immunoreactive perivascular astrocytes were taken from the striatum (**A**,**B**) and the internal capsule (**C**,**D**) with a 40× objective. The rectangles in the brain diagrams indicate the approximate locations where the photomicrographs were taken. Scale bar: 50 µm (for all panels).

In addition to the IgG extravasation, we found that GFAP-immunoreactive (GFAP+) hypertrophic astrocytes were closely associated with some vasculature in the BLG-sensitized mouse brains (Figure 12). Based on our qualitative examination of GFAP staining across the brain sections, we found that such perivascular astrogliosis was more commonly observed with larger blood vessels (Figure 12B,D) of the CMA mice. Furthermore, a greater number of hypertrophic astrocytes were present in cerebral white matter, where GFAP+ astrocytes were more abundantly located (Figure 12C,D). This result suggested that astrocytes were stimulated to alter vascular physiology and/or relay inflammatory signaling in our BLG-sensitized mice and that their activation was sustained by chronic exposure to the allergen.

### 3.6. Cortical Demyelination Was Evident in the BLG-Sensitized Mice after the Repeated Allergen Exposure

**Figure 13:**
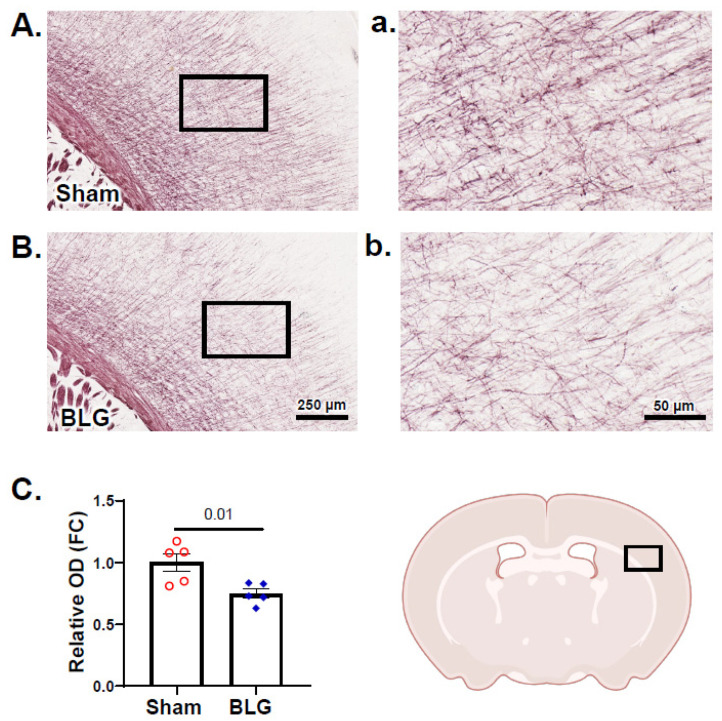
Histochemical evaluation and densitometric quantitation of Black Gold II myelin staining. Brain sections from the sham (**A**,**a**) and BLG-sensitized (**B**,**b**) mice were stained for myelin using Black Gold II. Photomicrographs were taken from the cerebral cortex at the level of the caudal striatum with 10× (**A**,**B**) and 40× (**a**,**b**) objectives. Scale bars: 250 µm (for (**A**,**B**)); 50 µm (for (**a**,**b**)). The rectangle in the brain diagram in the lower right indicates the approximate location where the photomicrographs were taken. The optical density (OD) of the staining was quantified using the QuPath software (**C**) and expressed as fold change (FC) calculated from the relative OD value of the sham mice. Each bar indicates the group average ± SEM (*n* = 5 per group). Red open circles and blue diamonds represent individual values for the sham and BLG mice, respectively. The numbers between the two bars indicate the *p*-values. Statistical significance (*p* < 0.05) was determined using Student’s *t*-test.

**Figure 14:**
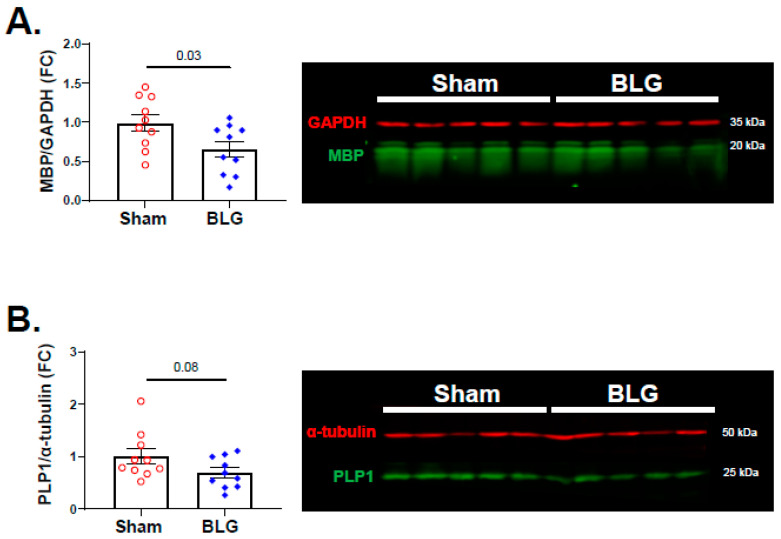
Quantitation of myelin proteins in the sham and BLG-sensitized mouse brains. The levels of myelin basic protein (MBP) (**A**) and proteolipid protein 1 (PLP1) (**B**) in the frontal cortex lysates were quantified using western blotting. The fluorescence signals from the MBP bands (green bands at 20 kDa) and the PLP1 bands (green bands at 25 kDa) were normalized respectively to GAPDH (red bands at 35 kDa) and α-tubulin (red bands at 50 kDa) in the corresponding lanes and expressed as fold change (FC) from the average value of the sham mice. Each bar indicates the group average ± SEM (*n* = 10 per group). Red open circles and blue diamonds represent individual values for the sham and BLG mice, respectively. The numbers between the two bars indicate the *p*-values. Statistical significance (*p* < 0.05) was determined using Student’s *t*-test.

MC proteases and increased BBB permeability have been associated with demyelination [59,60,61]. In addition to white matter astrogliosis described above, MC activation, histamine elevation, and IgG extravasation were observed in our whey-fed CMA mice, leading us to question whether myelin was also affected in these animals. When the brain sections were stained with Black Gold II, the myelin staining of large white matter structures, such as the corpus callosum and the internal capsule, appeared comparable between the sham group and the BLG-sensitized group (Figure S2). However, there was noticeably less staining in the motor and somatosensory regions of the cerebral cortex in the CMA mouse brains (Figure 13A,B). A quantitative comparison of the relative OD of the staining in the frontal cortex confirmed that myelin staining was indeed significantly lower in the BLG-sensitized mice (Figure 13C; sham: 1.0 ± 0.07; BLG: 0.8 ± 0.04; *p* = 0.01). Furthermore, western blotting of MBP, a major protein of the myelin sheath, was significantly decreased by almost 0.5-fold in the frontal cortex of the BLG-sensitized mice (Figure 14A; *p* = 0.03). PLP1, another structural protein of myelin, showed a decreasing trend, though it did not reach statistical significance (Figure 14B; *p* = 0.08). Together, our results demonstrated the presence of cortical demyelination in our mouse model of CMA after the repeated allergen exposure.

## 4. Discussion

There has been growing interest in the contribution of MCs to neuroinflammation and pathophysiology associated with neuropsychiatric and neurodegenerative disorders. However, the specific role of MCs in the development of these conditions has yet to be clarified. As a step toward understanding their involvement, we investigated intracranial MCs and associated neuropathologies in a mouse model of CMA, particularly when mice continued to be challenged repeatedly with the allergen.

Previously, we reported that BLG-sensitized mice developed hypersensitivity to the allergen with elevated IgE, although they were asymptomatic upon acute allergen challenge [47,48]. Interestingly, these observations were sex-dependent, and immunological and behavioral phenotypes of BLG-sensitized female mice were not significantly altered. In the present study, we, therefore, focused our investigation, using male mice only. We chose to challenge sensitized mice for a prolonged period with an allergen-containing diet to simulate repeated allergen consumption by asymptomatically sensitized individuals. This phenotype is referred to as “asymptomatic sensitization” [62] or “sensitized tolerance” [63] in human patients and, therefore, is of clinical importance. While individuals with life-threatening allergic reactions avoid offending allergens, sensitized but asymptomatic individuals can consume them without severe consequences, subjecting themselves to repeated allergen exposure. Since chronic inflammation is associated with various disease conditions [64,65,66,67,68], understanding the long-term consequences of immune activation is important for individuals with chronic immune disorders, such as allergies. Although female mice did not seem to be significantly affected by allergen sensitization and acute challenge in our previous studies [47,48], it is of our interest to include this experimental group and investigate whether prolonged allergen exposure would elicit delayed effects in females as distinct immunological and neurological phenotypes.

The BLG-sensitized mice were asymptomatic even after two weeks of the whey-containing diet. However, allergen-specific IgE was moderately elevated by week 5 and further increased during the allergen consumption (Figure 2A). By the end of week 7, it became several folds greater than in the respective sham group and our previous acute challenge models [47,48]. As the half-life of unbound IgE is 2–3 days [69], it was likely that the continuous allergen consumption further promoted IgE production. We also noted a slight increase in plasma IgE in the sham mice by the end of week 7 (Figure 2A). This small increase in IgE could have resulted from using CT in the vehicle during sensitization. Since CT increases intestinal permeability [70], it might have allowed transepithelial allergen entry during the whey diet period, even in the sham mice. This possibility could be confirmed by the inclusion of an additional set of sham and BLG-sensitized groups that would remain on the whey-free diet for the entire 7 weeks or histological analysis of intestines in future studies.

Despite the lack of typical allergy symptoms, the BLG-sensitized mice showed behavioral changes with the OFT during the second half of the recording period (Figure 3A,B,E and Figure S1). This time-dependent behavior in the second half of the OFT was not previously observed with our CMA model, in which BLG-sensitized mice were challenged with a single large dose of the allergen [48]. Furthermore, no mobility difference was observed between acutely challenged sham and BLG groups [47,48]. However, the chronically challenged sensitized mice exhibited a slower speed and a shorter travel distance, raising the possibility of motor deficits. This notion is further supported by the observation that the EZM results did not indicate anxiety-like behavior by the sensitized mice (Figure 4). In addition, the sensitized mice spent more time immobile during the TST (Figure 5A). Thus, this distinct behavioral phenotype may reflect motor impairment rather than anxiety, representing an outcome specific to repeated allergen consumption. Additional behavioral tests are required to directly assess motor function and further characterize the observed behavioral changes.

Irrespective of acute or chronic allergen exposure, intracranial MCs accumulated and/or were activated in the CMA mice (Figure 6 and Figure 7), suggesting that MCs were recruited to the CNS during sensitization. The increased prevalence of MCs persisted in the brain, choroid plexus, leptomeninges, and dura mater of the sensitized mice after repeated allergen consumption. The sustained presence of degranulated MCs led us to speculate that the allergen had triggered their activation through the IgE/FcεRI mechanism on MCs. Previously, IgE-mediated activation of intracranial MCs was questioned due to the lack of FcεRI expression by MCs [71]. However, MCs expressing FcεRI have been found in the brain parenchyma of rats with EAE [72]. Our results also indicated, at least in the dura mater, that intracranial MCs express FcεRI and that the receptor is primed with IgE in BLG-sensitized mice.

We also noted apparent differences in the staining density and morphologies of the FcεRI-immunoreactive MCs between the sham and BLG-sensitized mice (Figure 8). The more robust staining of MCs in the sham mice could be explained by the FcεRI antibody we used, which targeted the FcεRI alpha subunit, the IgE binding site [73]. Since dural MCs in the sensitized mice were strongly immunopositive for IgE, it is reasonable to postulate that the epitope on these MCs was not accessible to the FcεRI antibody due to the hindrance with bound IgE. Furthermore, the morphological difference of FcεRI-stained MCs in the BLG-sensitized mice may be explained by the property of MCs upon FcεRI activation. MCs, at least bone marrow-derived cultured MCs, have been shown to flatten and spread upon FcεRI-mediated activation for increased migratory activity [74]. Nonetheless, our results strongly suggested that intracranial MCs in the CMA mice were equipped to respond to the allergen via the IgE/FcεRI mechanism.

Thus, it is plausible that intracranial MCs in the CMA mice can be activated by allergens, particularly if the allergen has an ample opportunity to infiltrate intestines with repeated allergen consumption. Indeed, BLG was detected in the sera and the brain of the sensitized mice (Figure 9A,B), strengthening this possibility. However, regional differences in the levels of BLG were notable in the brain, with the midbrain region having a significantly greater amount of BLG than in the sham mice. The midbrain is a highly vascularized region, with the convergence of the major cerebral arteries, including the basilar, posterior cerebral, posterior communicating, and superior cerebellar arteries, supplying the region [75,76]. Therefore, the midbrain region may serve as an access point into the brain from circulation, and MCs in this region likely encounter BLG.

Contrary to the BLG levels, the midbrain region of the BLG-sensitized mice did not show a marked elevation in the histamine level (Figure 10A). In fact, the brain regions that showed elevated BLG levels (i.e., midbrain and striatal regions) did not have elevated histamine contents. The reason for this observation is unclear, though regional regulation of the central histaminergic system may be involved. Histamine is also a modulatory neurotransmitter in the brain, and its synthesis is regulated by histaminergic neurons in the tuberomammillary nucleus in the hypothalamic area [75,76,77]. In addition, their histaminergic fibers are widely distributed throughout the brain. Although intracranial MCs likely contributed to the altered brain histamine levels to some extent, additional investigation is required to assess the influence of MC-derived histamine on the regulation of brain-derived histamine.

The brain’s histaminergic system controls a variety of behaviors, including sleep–wake cycles, satiety, mobility, emotion, and memory (reviewed in [78]). The effects of histamine are mediated through four histamine receptor subtypes, H1–H4 receptors. Previously, we reported that greater H3R immunoreactivity was detected in the cortical region of whey protein-sensitized mice with acute allergen challenge, although brain histamine levels were not quantified in these mice [46]. In the present study, we found that H3R levels showed an increasing trend in the frontal cortex and significant elevation in the parietotemporal cortex, suggesting that the greater amounts of histamine found in these regions might have influenced the expression of the receptor. Since H3R functions as a presynaptic autoreceptor to regulate the activity of histaminergic neurons [79], regional increases in histamine concentration could have altered the receptor expression.

MC-derived histamine also controls vascular permeability, increasing transendothelial passage of leukocytes and other circulating factors as an initial step of inflammation [33,58]. In support of augmented brain MC activity in our CMA mice, we observed IgG extravasation (Figure 11), indicative of increased BBB permeability. The morphology of GFAP+ perivascular astrocytes (Figure 12) suggested their activation subsequent to BBB leakage in their vicinity (see review [80]). In addition, we detected readily noticeable histological evidence for cortical demyelination (Figure 13), which was further validated by quantifying MBP (Figure 14A). Since MC proteases have been shown to degrade myelin proteins in vitro [61], CMA-associated MC activation could have resulted in demyelination in this region. In addition to histamine levels, we also detected significantly elevated levels of MCPT-1 in the frontal and parietotemporal cortices (Figure S3), supporting this hypothesis. Alternatively, elevated histamine might have affected oligodendrocytes since the myelin-forming glial cells express H3R, and their differentiation is partly regulated by the receptor activation [81]. The causative role of MCs in CMA-associated cortical demyelination warrants further investigation as the involvement of MCs in multiple sclerosis, a demyelinating disease, has become increasingly evident [77,82,83]. It would also be of future interest to determine whether the demyelination in BLG-sensitized mice is reversible upon discontinuation of allergen consumption.

## 5. Conclusions

Based on our results, we conclude that MCs are recruited to the CNS during sensitization and become activated by the allergen that enters the circulation. Furthermore, intracranial MCs are equipped with the IgE/FcεRI-mediated degranulation mechanism. Prolonged or repeated allergen exposure likely sustains MC activation, BBB impairment, and neuroinflammation, promoting neurodegenerative events. Further investigation is required to validate the causative role of MC mediators in the development of neuropathologies and behavioral alterations. The outcomes of this study also suggest that continued consumption of offending allergens could maintain inflammatory status in sensitized individuals even in the absence of severe allergic symptoms.

## Figures and Tables

**Table 1:** Primary antibodies used for western blotting.

Antibody	Host	Clonality	Product No.	Dilution	Company
α-tubulin	Mouse	Monoclonal	sc-8035	1:1000	Santa-Cruz Biotechnology, Inc.
Glyceraldeyde-3-phosphate (GAPDH)	Mouse	Monoclonal	sc-32233	1:1000	Santa-Cruz Biotechnology, Inc.
Histamine H3 receptor (H3R)	Rabbit	Polyclonal	AHR-003	1:500	Alomone Labs
Myelin basic protein (MBP)	Rabbit	Polyclonal	PA5-78397	1:5000	Invitrogen, Thermo Fisher Scientific
Mast cell protease-1 (MCPT-1)	Rabbit	Monoclonal	MA5-38007	1:500	Invitrogen, Thermo Fisher Scientific
Proteolipid protein 1 (PLP1)	Rabbit	Polyclonal	PA3-150	1:500	Invitrogen, Thermo Fisher Scientific

## Data Availability

The data presented in this study are available on request from the corresponding author.

## Supplementary Materials

The following supporting information can be downloaded at: www.mdpi.com/article/10.3390/cells11040738/s1, Figure S1: Mobility and anxiety-like behavior assessed with OFT in 1-min intervals; Figure S2: Black Gold II histochemical staining of large white matter structures; Figure S3: Quantitation of MCPT-1 in the cortical regions of sham and BLG-sensitized mice; Figure S4: Uncropped H3R and GAPDH near-infrared western blots; Figure S5: Uncropped MBP and GAPDH near-infrared western blots; Figure S6: Uncropped PLP1 and α-tubulin near-infrared western blots; Figure S7: Uncropped MCPT-1 and α-tubulin near-infrared western blots.
