# Supplementary material for: Potential Role of Intracranial Mast Cells in Neuroinflammation and Neuropathology Associated with Food Allergy
Source: Cells. 2022 Feb 20;11(4):738. doi: 10.3390/cells11040738 (PMC8870724; doi:10.3390/cells11040738)
Supplement: Supplementary file 1 [file cells-11-00738-s001.zip › cells-1558856-supplementary.pdf]

Supplementary Figures

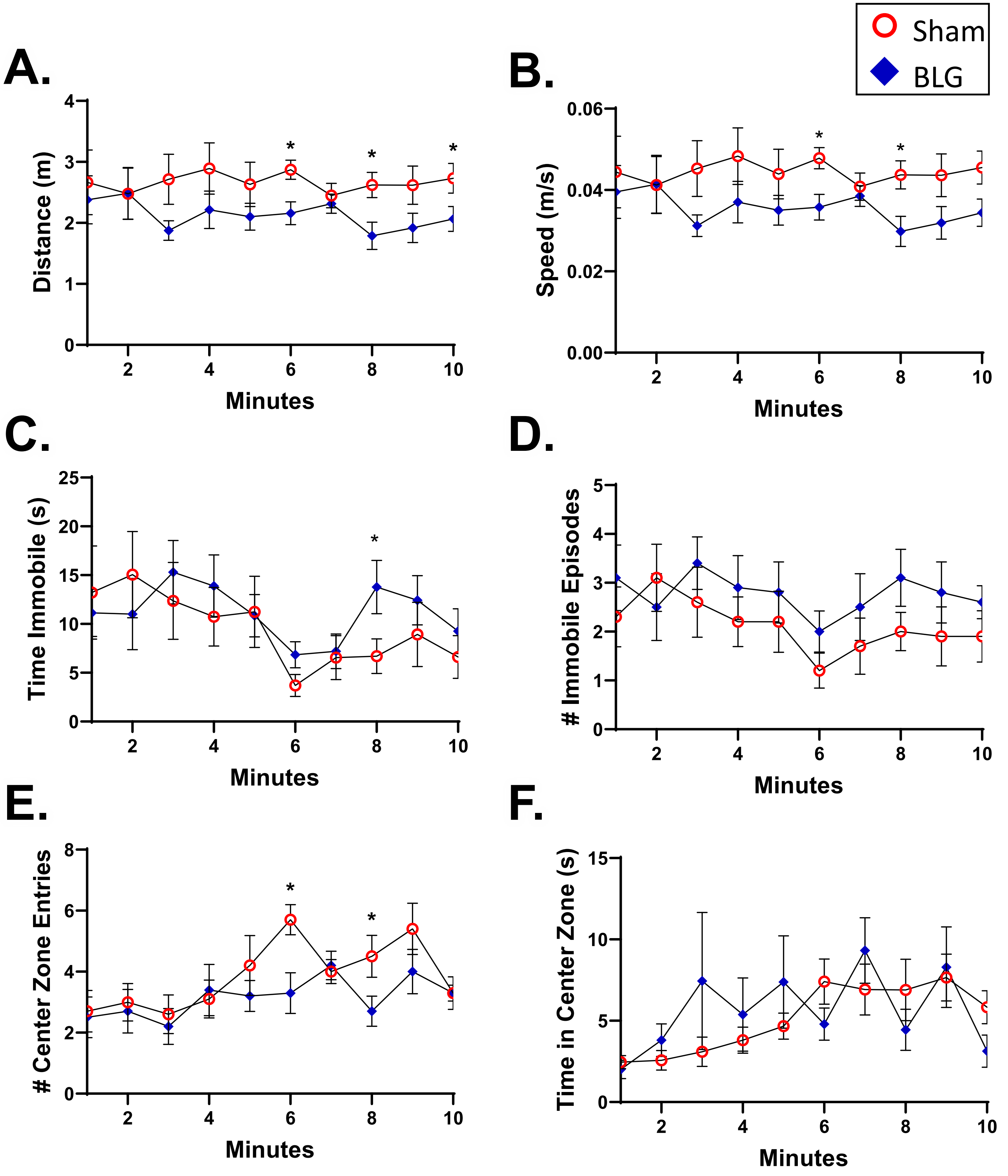

Figure S1: Mobility and anxiety-like behaviors assessed with OFT in 1-min intervals. Behavioral parameters measured with the OFT, including distance (**A**), speed (**B**), total time immobile (**C**), number of immobile episodes (**D**), number of center zone entries (**E**), and time spent in the center zone (**F**), were plotted at 1-min intervals. Red open circles and blue diamonds represent the average values at each time point for sham and BLG mice, respectively (n=10 per group). Asterisks denote significantly significant differences between the groups at the given time point. Statistical significance ($p < 0.05$) was determined by multiple uncorrected t-tests.

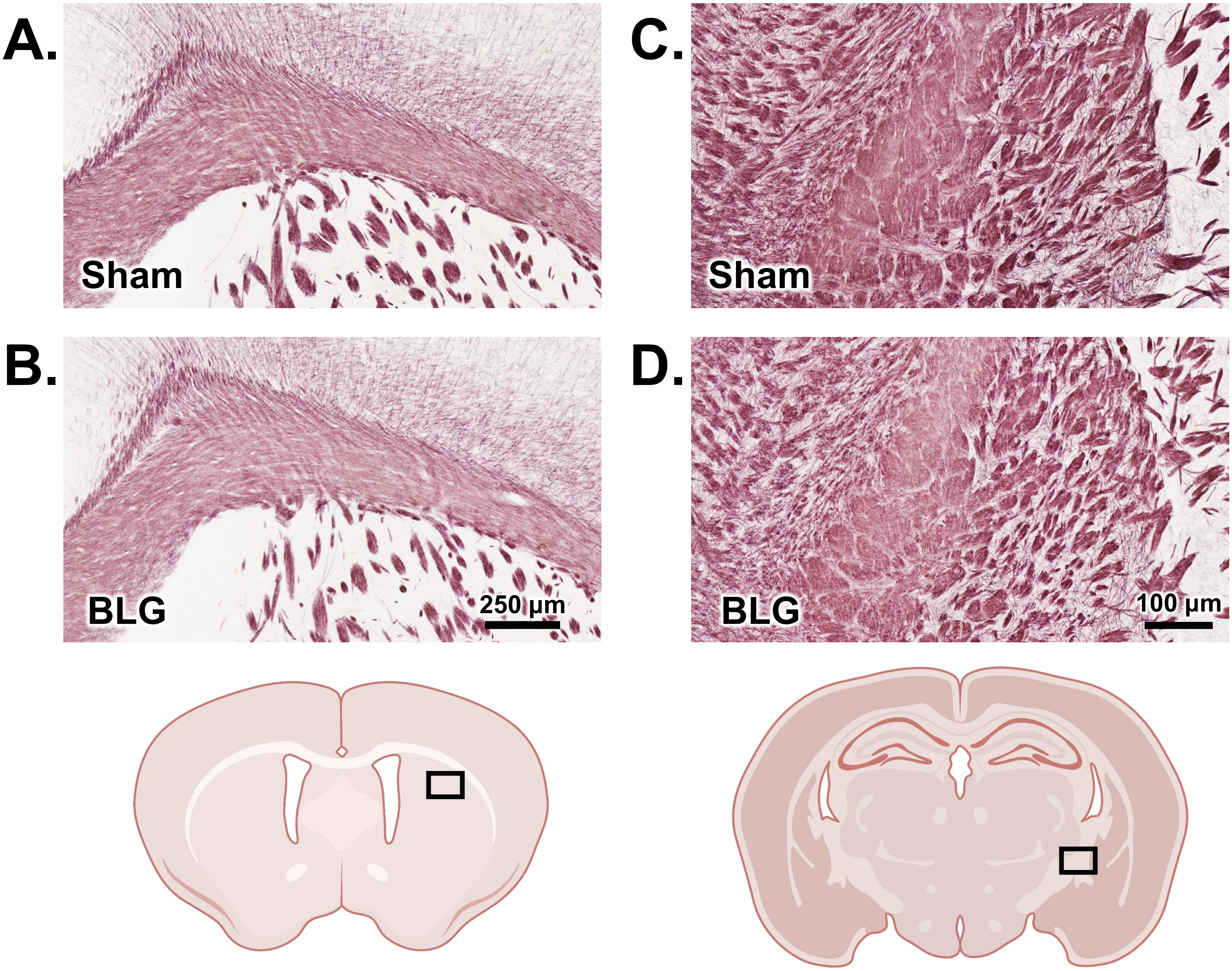

Figure S2: Black Gold II histochemical staining of large white matter structures. Brain sections from sham and BLG-sensitized mice were stained with Black Gold II. Areas including the corpus callosum (**A & B**) and internal capsule (**C & D**) are shown. The rectangles in the brain diagrams denote the areas where the photomicrographs in panels A-D were taken. Photomicrographs were taken with 10x (A & B) and 20x (C & D) objectives. Scale bars: 250 µm (for A & B); 100 µm (for C & D).

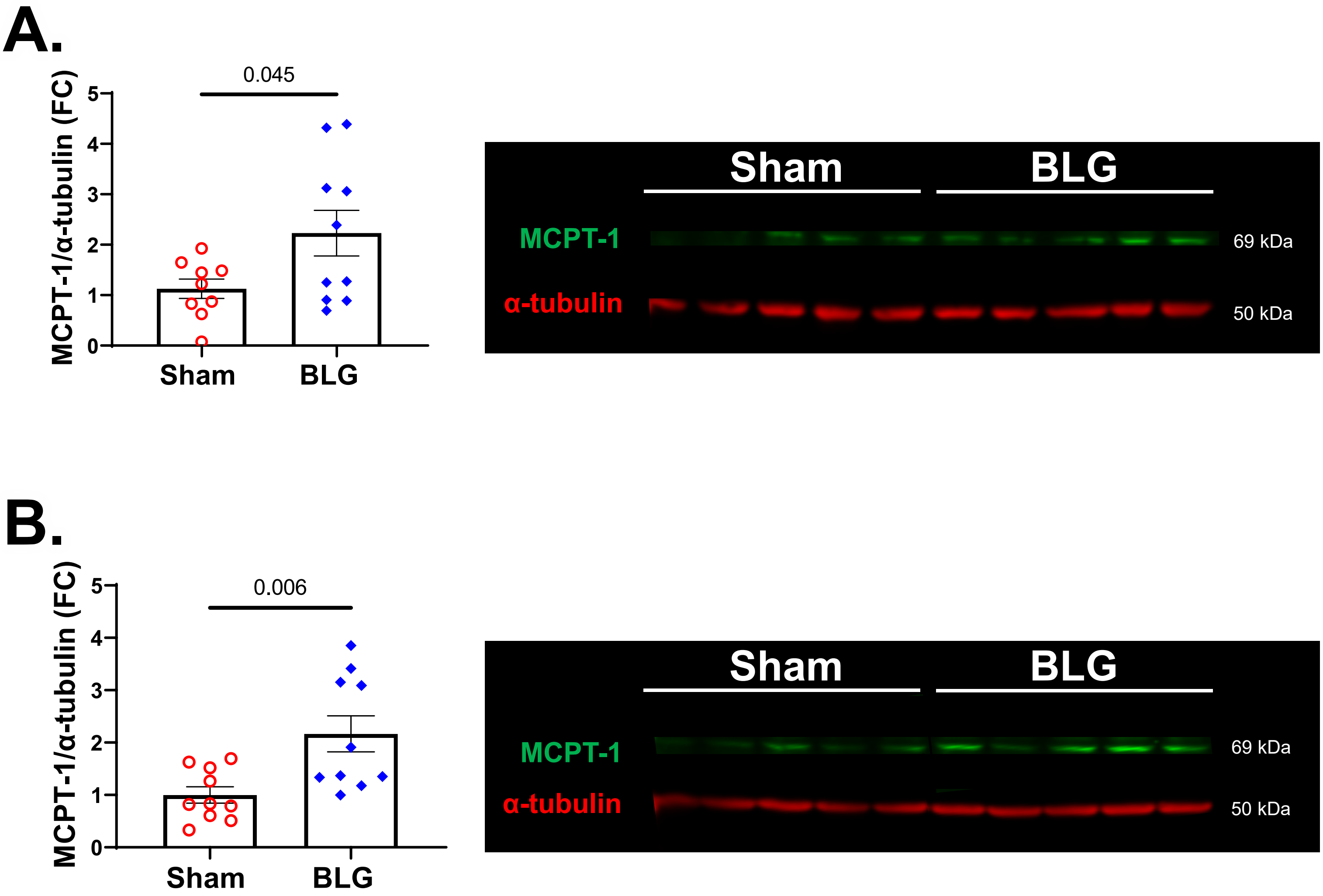

Figure S3: Quantitation of MCPT-1 in the cortical regions of sham and BLG-sensitized mice. The levels of MCPT-1 in the lysates prepared from the frontal cortex (**A**) and parietotemporal cortex (**B**) were quantified using western blotting. The fluorescence signals from the MCPT-1 bands (green bands at 69 kDa) were normalized to α-tubulin (red bands at 50 kDa) in the corresponding lanes and expressed as fold change (FC) from the average value of sham mice. Each bar indicates the group average ± SEM (n=10 per group). Red open circles and blue diamonds represent individual values for sham and BLG mice, respectively. The numbers between two bars indicate the *p*-values. Statistical significance ($p < 0.05$) was determined by Student's t-test**.**

Original Blots

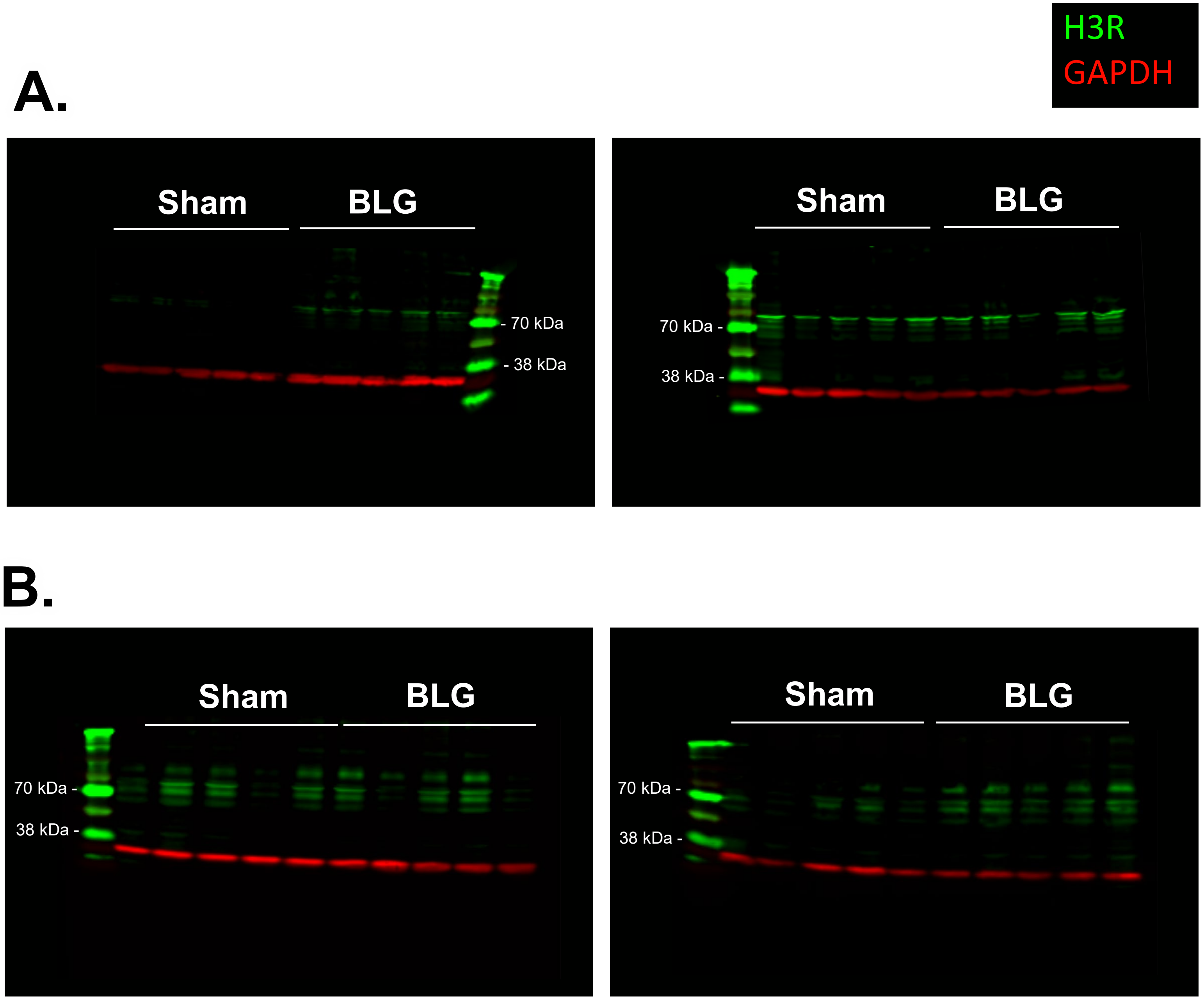

Figure S4: Uncropped H3R and GAPDH near-infrared western blots. Twenty µg of protein preparations from the frontal cortex (**A**) and parietotemporal cortex (**B**) of sham and BLG-sensitized mice were resolved on two 10% polyacrylamide gels (n=10 per group). Green bands at ~75 kDa and red ands at ~35 kDa represent H3R and GAPDH proteins, respectively.

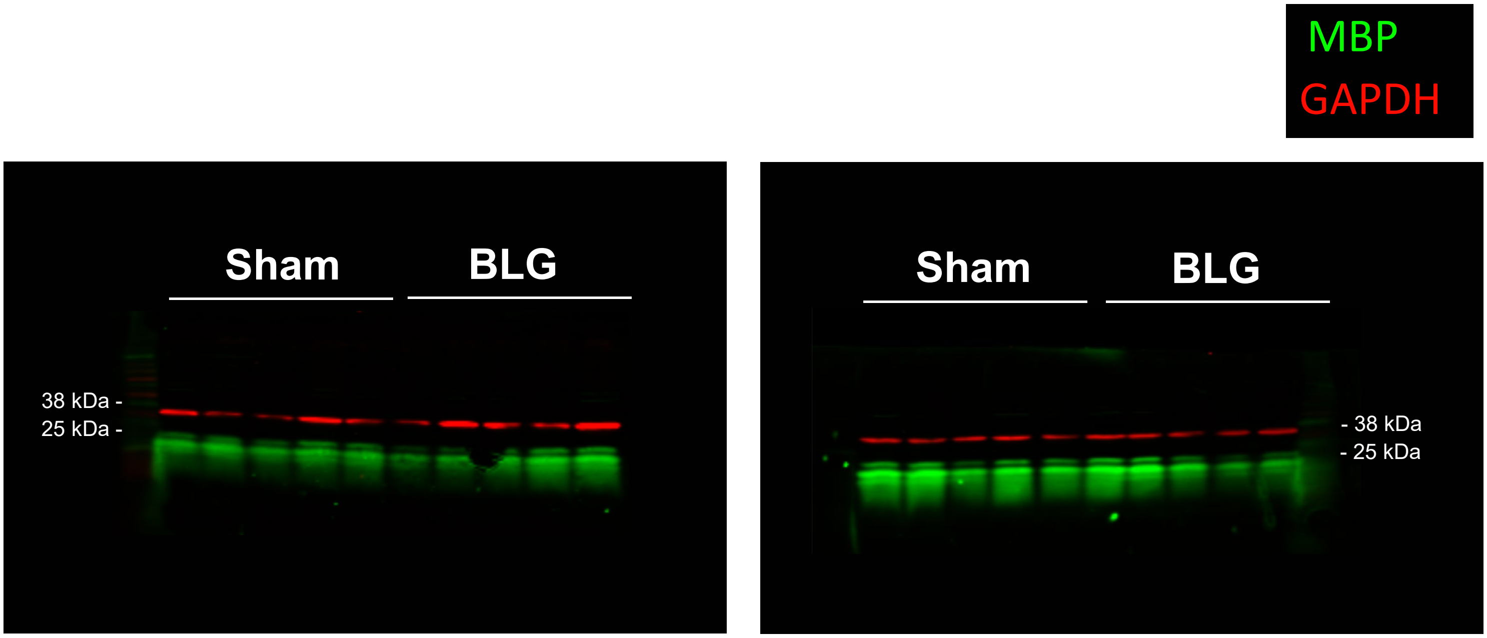

Figure S5: Uncropped MBP and GAPDH near-infrared western blots. Twenty µg of protein preparations from the frontal cortex of sham and BLG-sensitized mice were resolved on two 15% polyacrylamide gels (n=10 per group). Green bands at ~20 kDa and red bands at ~35 kDa represent MBP and GAPDH proteins, respectively.

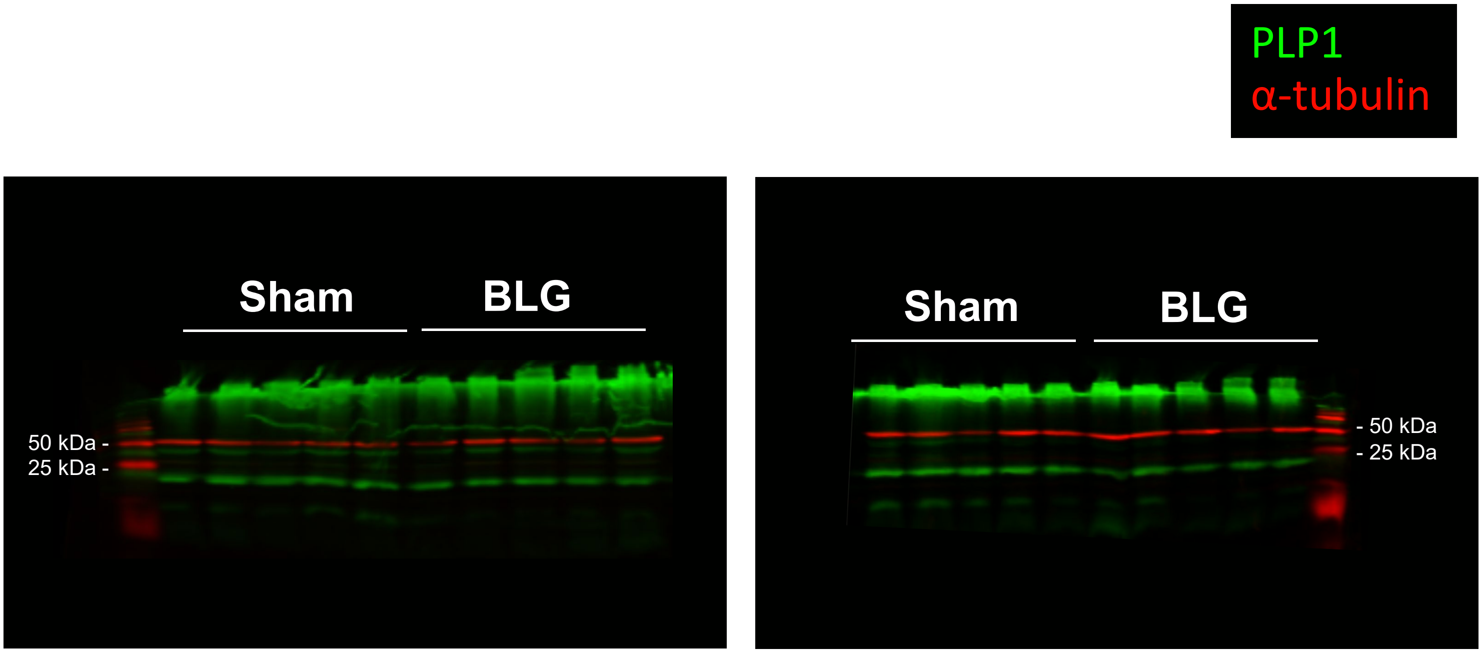

Figure S6: Uncropped PLP1 and α-tubulin near-infrared western blots. Twenty µg of protein preparations from the frontal cortex of sham and BLG-sensitized mice were resolved on two 15% polyacrylamide gels (n=10 per group). Green bands at ~25 kDa and red bands at ~50 kDa represent PLP1 and α-tubulin proteins, respectively.

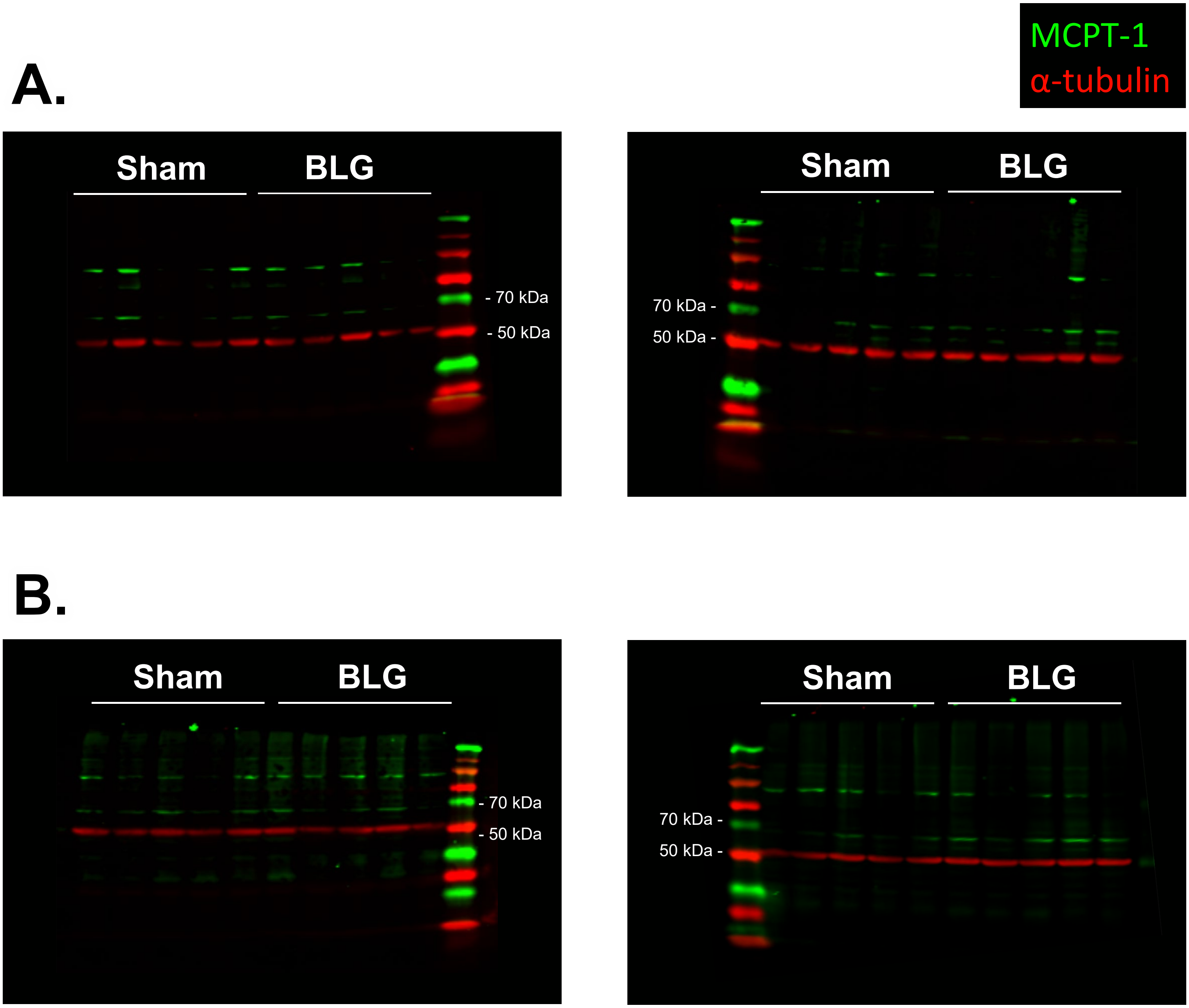

Figure S7: Uncropped MCPT-1 and α-tubulin near-infrared western blots. Twenty μg of protein preparations from the frontal cortex (**A**) and parietotemporal cortex (**B**) of sham and BLG-sensitized mice were resolved on two 10% polyacrylamide gels (n=10 per group). Green bands at ~69 kDa and red bands at ~50 kDa represent MCPT-1 and α-tubulin proteins, respectively.
